# Binding of 14-3-3 stabilises recombinant AMPKγ2-containing complexes

**DOI:** 10.1042/BCJ20250342

**Published:** 2026-04-13

**Authors:** Shu-Yang Chen, Jane Bennett, Naveenan Navaratnam, Rebeca Fiadeiro, Angela Woods, Alex Montoya, Pavel V. Shliaha, Simone Kunzelmann, Steven A. Howell, Shahid Mehmood, Andrew G. Purkiss, Jon R. Wilson, Steven J. Gamblin, David Carling

**Affiliations:** 1 Cellular Stress Group, MRC Laboratory of Medical Sciences, Hammersmith Hospital, Imperial College, London W12 0HS, U.K.; 2 Proteomics Facility, MRC Laboratory of Medical Sciences, Hammersmith Hospital, Imperial College, London W12 0HS, U.K.; 3 Structural Biology Science Technology Platform, Francis Crick Institute, 1 Midland Road, London NW1 1AT, U.K.; 4 Proetomics Structural Biology Science Technology Platform, Francis Crick Institute, 1 Midland Road, London NW1 1AT, U.K.; 5 Francis Crick Institute, 1 Midland Road, London NW1 1AT, U.K.; 6 Institute of Clinical Sciences, Faculty of Medicine, Hammersmith Hospital, Imperial College, London W12 0NN, U.K.

**Keywords:** 14-3-3 proteins, AMPK, phosphorylation/dephosphorylation

## Abstract

AMP-activated protein kinase (AMPK) plays an important role in maintaining energy homeostasis in mammals. AMPK is a heterotrimer of an α catalytic subunit and two regulatory subunits, β and γ. In mammals, each subunit has different isoforms (α1/α2, β1/ β2, and γ1/γ2/γ3) encoded by separate genes, leading to the potential expression of 12 AMPK complexes. Here, we show that AMPK containing the long forms of γ2 (γ2a, encoding a protein of 569 amino acids, and γ2c, 525 amino acids) binds to 14-3-3. In contrast to AMPK containing the short form of γ2 (γ2b, 328 amino acids), bacterial expression of AMPK containing the long forms of γ2 requires co-expression with 14-3-3 and prior phosphorylation of Thr172 within the α subunit. AMPKγ2-14-3-3 complexes have reduced activity compared with AMPKγ1 or AMPKγ2b but retain allosteric activation by AMP and the AMPK activator, 991. We found that two predicted 14-3-3 binding sites within γ2a (T97 and S122) were phosphorylated in the bacterially expressed AMPK complex. Furthermore, we show that a peptide spanning these two phosphorylated sites binds to 14-3-3 *in vitro* and determined the crystal structure of this 14-3-3-peptide co-complex. These results indicate that 14-3-3 binds to the N-terminal region of γ2a/c, reducing the activity of AMPK relative to AMPKγ1 and AMPKγ2b. Our findings reveal a new mode of regulation of AMPK containing the long forms of γ2. While the biological significance of 14-3-3 binding to AMPKγ2a/c complexes remains to be determined, our studies provide the starting point to begin to address this issue.

## Introduction

Mammalian AMP-activated protein kinase (AMPK) complex consists of a catalytical subunit (α) that contains a canonical kinase domain and two regulatory subunits (β and γ) [1]. A key feature of AMPK is that its activity depends on the relative levels of ATP, ADP, and AMP within the cell [2]. When cellular ATP levels are high relative to ADP and AMP, AMPK exists in a low activity state. In contrast, if ATP levels fall, and the intracellular ATP:ADP and ATP:AMP ratios fall, AMPK activity increases. This activation is brought about by an increase in the phosphorylation of threonine 172 (Thr172) within the activation loop of the α subunit, as well as by allosteric activation by AMP [3]. Once activated, AMPK phosphorylates its target substrates, causing a change in a wide and varied number of pathways within the cell, and recent estimates indicate that AMPK can phosphorylate more than 100 substrates [4]. Overall, the combined effect of changes in AMPK substrate phosphorylation is to lower ATP-utilising anabolic pathways, e.g., fatty acid and cholesterol synthesis, and to increase pathways that lead to ATP production, e.g., fatty acid oxidation [1,4–8]. In addition to these acute effects, AMPK activation has longer-term effects on the cell, including changes in mitochondrial function [6] and cell proliferation [5,9].

The γ subunit harbours four cystathionine-β-synthase (CBS) domains, termed CBS1–CBS4 [10]. Structural and biochemical studies have revealed that CBS4 is permanently occupied with a non-exchangeable AMP molecule, whereas CBS2 is unoccupied [11]. CBS1 and CBS3 bind adenine nucleotides in an exchangeable and competitive manner, and so these sites can act as adenine nucleotide sensors [11–13]. Previous studies have shown that binding to CBS3 plays the key role in regulating AMPK activity in response to changes in adenine nucleotides [12]. In humans, there are three genes encoding AMPK γ subunit isoforms (*PRKAG1*, encoding γ1; *PRKAG2*, encoding γ2; and *PRKAG3*, encoding γ3) [1]. The γ1 and γ2 isoforms are broadly expressed in mammalian cell types, with the γ1 being the predominant isoform in most tissues [14]. Expression of the γ3 isoform is almost exclusively restricted to fast-twitch skeletal muscle fibres [14], although a recent study reported expression of γ3 in a subset of mature oligodendrocytes from aged mice [15].

**Figure 1:**
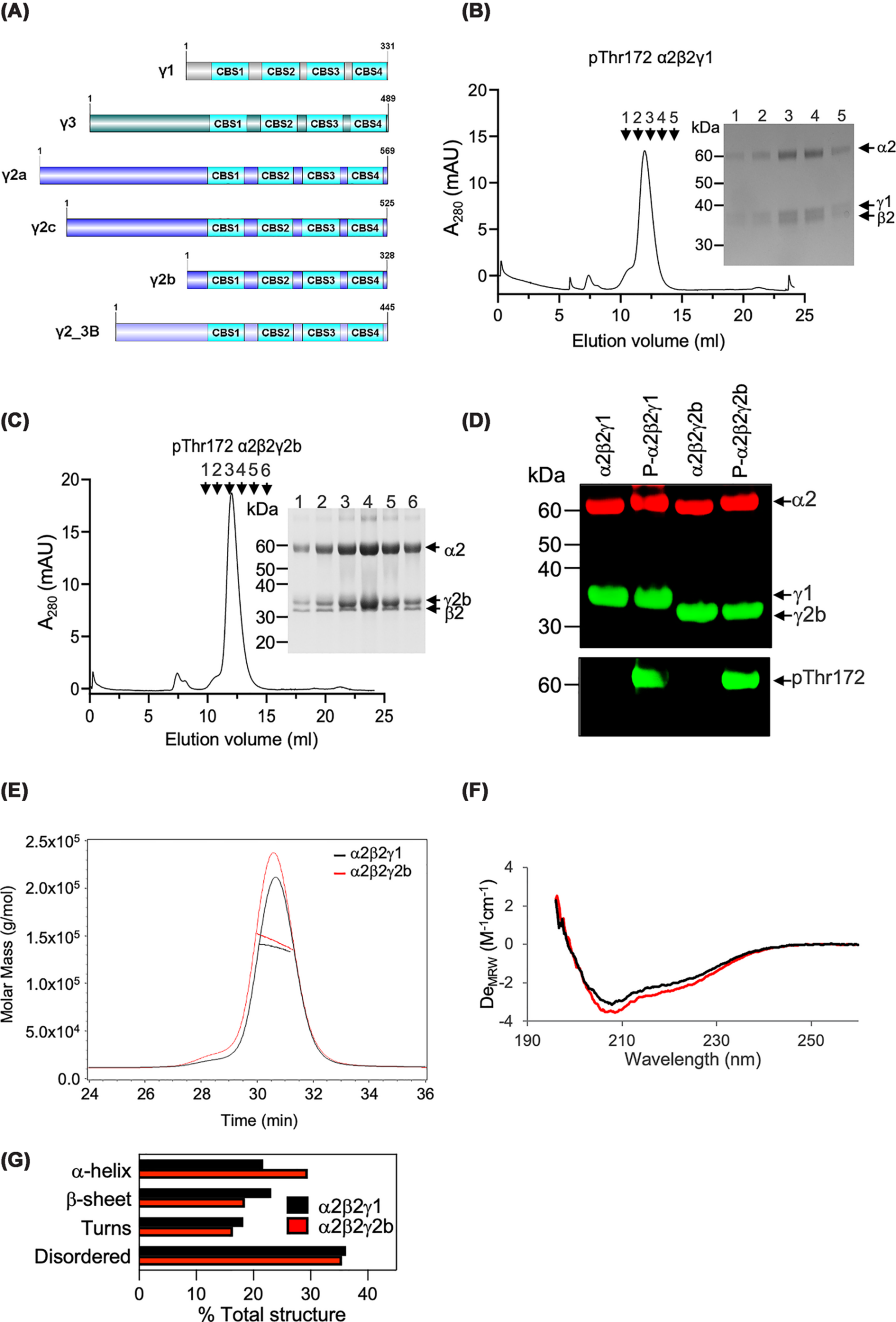
Characterisation of recombinant AMPKγ1 and AMPKγ2b complexes (**A**) Cartoon of AMPK γ isoforms showing conserved CBS domains and variable N-terminal regions. (**B,C**) Elution profiles (absorbance at 280 nm in milli-absorbance units (mAU)) following gel filtration of recombinant AMPKα2β2γ1 (**B**) and AMPKα2β2γ2b (**C**) complexes. In both cases, AMPK complexes were co-expressed with CAMKK2 to allow phosphorylation of Thr172 within the α subunit of AMPK. Aliquots of the indicated fractions were analysed by SDS–PAGE, and Coomassie-stained gels are shown (inset). Migration of molecular mass standards is indicated, and arrows identifying the bands corresponding to α2, β2, γ1, and γ2b are shown. (**D**) Western blot analysis of the peak protein fraction following gel filtration for non-phosphorylated and phosphorylated Thr172 AMPKα2β2γ1 and AMPKα2β2γ2b complexes. Blots were probed with mouse anti-α1/2 (shown in red), rabbit anti-pThr172, rabbit anti-γ1, rabbit anti-γ2 and rabbit anti-β2 antibodies (all shown in green). (**E**) SEC-MALS (size exclusion chromatography-multi-angle light scattering) spectra for the molar mass distributions of AMPKγ1 and AMPKγ2b complexes. (**F**) Far-UV CD spectra of AMPK complexes. Measurement of the molar ellipticity in degrees (De_MRW_) was carried out at 20°C. (**G**) Protein secondary structure content (% total) from CD data was predicted using open-source software CDPro.

All three γ-isoforms contain a highly conserved C-terminal domain consisting of four CBS repeats that bind adenine nucleotides (see Figure 1A) [4,8]. Compared with the human γ1 isoform, which has a short N-terminal region of 37 amino acids (up to a conserved cysteine residue in CBS1 of the human γ isoforms), the γ3 isoform has a long N-terminal domain of up to 192 amino acids that shares no obvious sequence homology with other proteins [4,8]. Four transcriptional variants of γ2 (a, b, c, and 3B) have been identified encoding proteins that differ in their N-terminal region (Figure 1A and Supplementary Figure S1A) [16]. The γ2a variant encodes the long form of γ2 (569 amino acids), while γ2b encodes the short form of γ2 (328 residues lacking the first 241 amino acids of γ2a) [16]. Transcription of a third variant (γ2c) starts in exon 2, encoding a protein of 525 amino acids that is identical to γ2a but lacking the first 44 amino acids [16]. A fourth variant, γ2-3B, encodes a protein of 445 residues, with a unique N-terminal sequence of 32 amino acids [16]. While data examining the expression of the different variant transcripts is available from the Genotype-Tissue Expression Portal, to our knowledge, there is currently very little information available at the level of protein expression for the different variants. In one study, antibodies reported to be specific for the γ2-3B variant indicate the highest expression in the heart, followed by the brain and liver, with undetectable expression in skeletal muscle and kidney [16]. In the same study, using an antibody that is reported to cross-react with all variants, evidence is presented suggesting that the γ2-3B variant is the predominant form expressed in the cytoskeletal fraction from mouse heart [16]. In another study, using an antibody claimed to be specific for γ2a and γ2c, the γ2a variant is reported to be more highly expressed in a non-failing human heart [17]. It should be noted that the details of the antibodies used in both of these studies are incomplete, and robust data validating the selectivity of the antibodies is lacking, and so caution should be used when interpreting the results.

The N-terminal region of the long form of γ2 is predicted to be disordered (Supplementary Figure S1B). Naturally occurring mutations within the conserved C-terminal region of γ2 containing the 4 CBS domains have been identified in humans that lead to a cluster of severe cardiac abnormalities, including cardiac hypertrophy, glycogen storage, and ventricular pre-excitation (Wolff–Parkinson–White syndrome) [1,18]. We reported previously that in cell-based studies, the small molecule AMPK activator 991 leads to a greater increase in T172 phosphorylation in complexes containing the long form of γ2 compared with complexes containing either γ1 or γ3 [19]. Furthermore, we showed that this effect was dependent on the N-terminal region of γ2 [19]. The mechanistic basis for this effect is unclear, and whether the N-terminal region of the long forms of γ2 plays any physiological role in regulating AMPK remains unclear. In this study, we identify two sites within the N-terminal regions of γ2a and γ2c that when phosphorylated bind to 14-3-3. Co-expression of the AMPK α2β2γ2c complex with 14-3-3 in *Escherichia coli* allows purification of a stable recombinant AMPKγ2/14-3-3 co-complex. The AMPK/14-3-3 co-complex has reduced activity compared with γ1 and γ2b AMPK complexes. Our study provides a foundation for investigating the physiological function of the AMPKγ2a/c complexes in association with 14-3-3.

## Results

### The unstructured N-terminal region of the long form of γ2 isoform causes instability of the AMPK complex

To date, the role of the extended N-terminal region of the long forms of γ2 (γ2a/c) has not been characterised. To address this gap, we expressed AMPK complexes harbouring different γ2 variants in *E. coli* using the strategy previously established for expression of the AMPKγ1 complex (as detailed in the ‘Materials and methods’ section) [20,21]. Using this approach, we were able to express and purify the recombinant AMPKγ2b complex (the short form of γ2) to a similar degree of purity and yield as that obtained with the AMPKγ1 complex. Notably, the AMPKγ2b and AMPKγ1 complexes exhibited similar profiles for the final size exclusion chromatography (SEC) step, as well as in subunit characterisation following SDS–PAGE (Figure 1B,C). Importantly, like the AMPKγ1 complex, AMPKγ2b protein was efficiently expressed either in the phosphorylated or non-phosphorylated Thr172 forms, as evidenced by Western blot analysis of the purified complexes (Figure 1D). SEC-MALS analysis confirmed that both AMPK complexes are highly homogeneous heterotrimers (Figure 1E), and circular dichroism (CD) revealed that they have similar secondary structure contents between them (Figure 1F,G).

In contrast, we were unable to purify stable, homogeneous complexes of AMPKγ2a or AMPKγ2c using the same approach. These AMPKγ2 complexes were eluted in the void volume of SEC, indicating that they formed aggregates (Supplementary Figure S2). SDS–PAGE and Western blot analysis of the expressed proteins revealed significant degradation of the N-terminal region of the γ2 subunit (detected by probing with an antibody recognising the C-terminus of the γ2 subunit) (Supplementary Figure S2). Despite extensive modification of expression conditions, purification buffers, and affinity tags, we were unable to improve the solubility and stability of these AMPKγ2 complexes (see the ‘Materials and methods’ section and Supplementary Table S1 for more details). A likely explanation for the inability to purify these recombinant proteins is that the disordered γ2 N-terminal extensions contribute to the instability of the AMPK complexes. This presents a major challenge for subsequent biochemical, biophysical, and structural analyses of these complexes. We noted that the N-terminal extension of the long forms of γ2 contains three potential phosphorylation sites (T97 (T53 in γ2c), S122 (S78 in γ2c), and S131 (S87 in γ2c)) that are highly scored as 14-3-3 binding sites using the prediction tool 14-3-3-Pred [22]. The amino acid sequences spanning these phosphorylation sites are highly conserved across mammalian species, with the exception that T97 is substituted for a methionine residue in rat (Supplementary Figure S1C). The 14-3-3 protein family consists of seven members (beta (β), epsilon (ε), eta (η), gamma (γ), tau (τ), sigma (σ), and zeta (ζ)), encoded by distinct genes, that are highly conserved and widely expressed in mammalian cells [23,24]. 14-3-3 proteins form homo- and heterodimers that bind to a wide range of proteins, primarily through phosphorylation-dependent interactions, and the dimeric 14-3-3 protein can potentially bind to two phosphorylation sites through each monomer.

### Bacterially expressed AMPKγ2a/c forms a stable co-complex with 14-3-3ε

**Figure 2:**
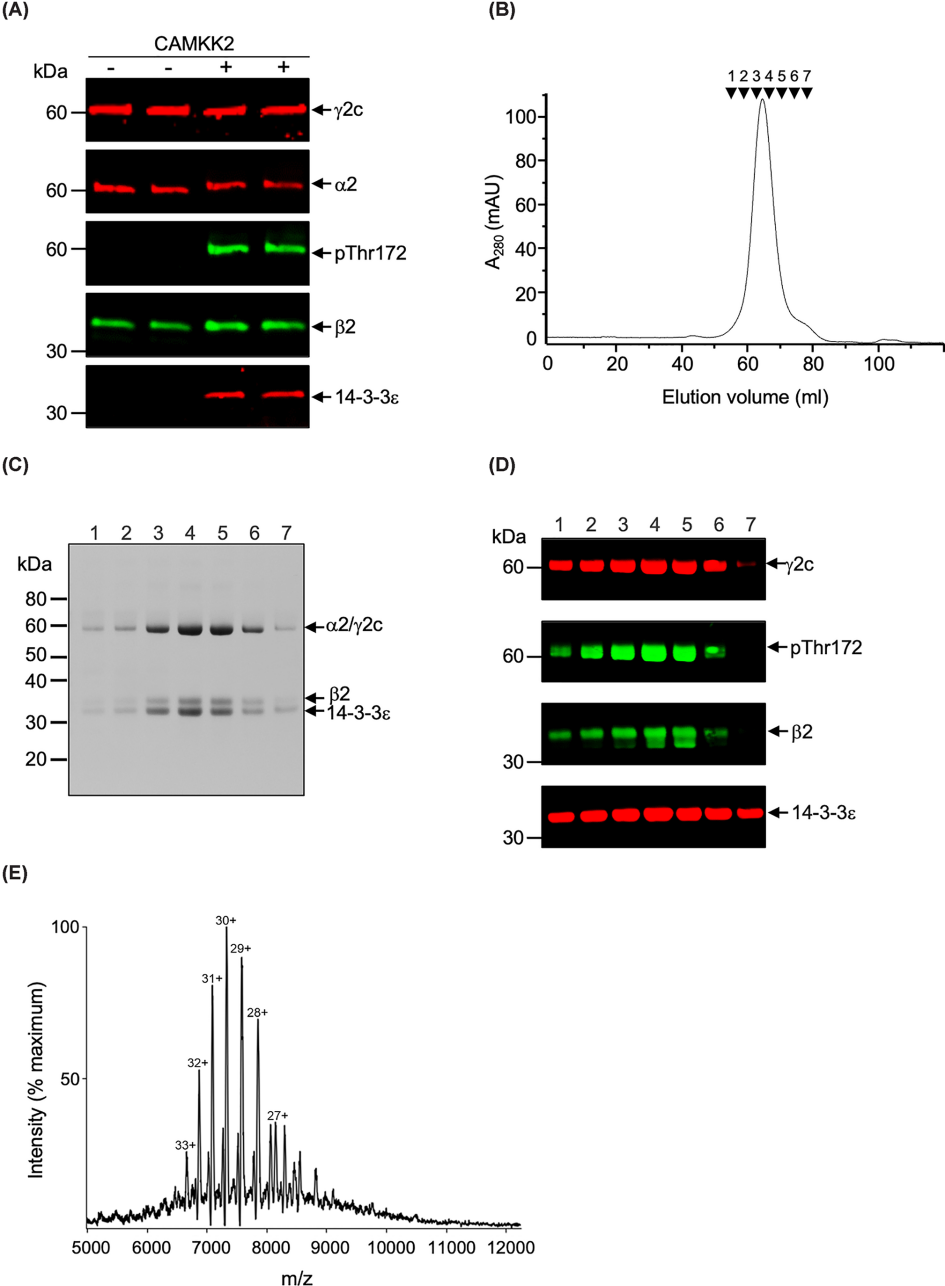
Purification and characterisation of recombinant AMPKγ2c/14-3-3ε co-complex protein expressed in bacteria (**A**) AMPKα2β2γ2c and 14-3-3ε were co-expressed in bacteria with or without co-expression of CAMKK2. Cell lysates were incubated with Strep-Tactin XT Sepharose resin, and bound proteins eluted with buffer containing 3 mM d-desthiobiotin (the γ2c construct contains an N-terminal Strep-tag). The eluted proteins were analysed by Western blotting as indicated. (**B**) Elution profile of AMPKγ2c (pThr172 form)/14-3-3ε co-complex following gel-filtration. Aliquots from the fractions indicated by the arrows were analysed by SDS–PAGE (**C**) and western blotting (**D**). Migration of molecular mass standards is indicated, and arrows identifying the bands corresponding to α2, β2, γ2c, and 14-3-3ε are shown. Note that α2 and γ2c co-migrate. A mouse anti-Strep antibody was used to detect γ2c (shown in red). (**E**) Native mass spectrum of the AMPKγ2c/14-3-3ε co-complex.

To determine whether 14-3-3 could bind and stabilise the γ2 N-terminal extension, we designed a strategy for co-expression of AMPKα2β2γ2c with 14-3-3 in bacteria (described in the 'Materials and methods' section). We used the AMPKα2β2γ2c complex, as our initial studies indicated that this complex was expressed in *E. coli* at substantially higher levels than the AMPKα2β2γ2a complex. As shown in Figure 2, α2β2γ2c co-purified with 14-3-3ε following affinity chromatography purification when co-expressed with Ca^2+^/calmodulin-dependent kinase kinase 2 (CAMKK2). CAMKK2 phosphorylates Thr172 within the activation loop of the α subunit, activating AMPK [3]. In the absence of CAMKK2, there was no detectable binding of 14-3-3 to AMPK (Figure 2A). In contrast, AMPK phosphorylated on Thr172 eluted in a symmetrical peak in the final gel-filtration step (Figure 2B). SDS–PAGE and western blot analysis of fractions from the elution peak confirmed that the co-complex consists of all three AMPK subunits, together with 14-3-3ε (Figure 2C,D). We assessed the stoichiometry of the co-complex using native mass spectrometry (Figure 2E), which indicated that the dominant species in the purified co-complex had a molecular mass of ∼217 kDa, consistent with the predicted molecular mass of AMPKα2β2γ2c heterotrimer bound to a 14-3-3ε dimer. We investigated the sites phosphorylated in γ2 in the recombinant AMPK co-complex by mass spectrometry following digestion by either trypsin or chymotrypsin. Using this approach, we were able to identify phosphorylation of T97 and S122 but not S131 (numbering of residues refers to γ2a; Supplementary Figure S3). Furthermore, AMPK, but not CAMKK2, was able to phosphorylate a synthetic peptide based on the sequence of γ2a spanning Thr97 and Ser122 (Supplementary Figure S3E). Taken together, these findings suggest that AMPK is able to autophosphorylate γ2 on T97 and S122, and that this allows binding of 14-3-3, which stabilises the AMPK complex.

### *In vitro* binding studies with 14-3-3

**Figure 3:**
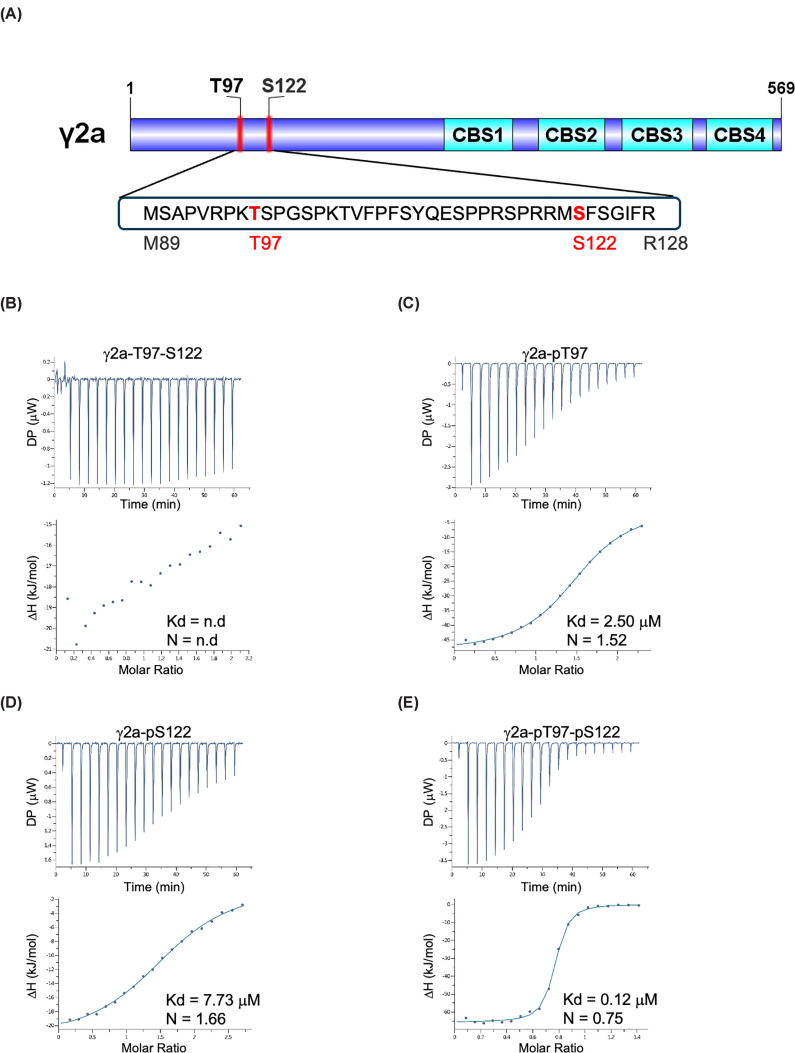
14-3-3 binding to a peptide spanning T97 and S122 in γ2a (**A**) Cartoon showing the sequence surrounding T97 and S122 in the N-terminal extension of γ2. Four peptides spanning residues M89-R128 of γ2a were synthesised: a non-phosphorylated peptide (γ2a-T97-S122), two singly phosphorylated peptides (γ2a-pT97 and γ2a-pS122) and a doubly phosphorylated peptide (γ2a-pT97-pS122). (**B**–**E**) Isothermal calorimetry experiments showing binding curves for the different peptides. In each case, 20 μM 14-3-3ε dimer was titrated with 400 μM peptide at 20°C to determine the dissociation constant (*K*_d_) and stoichiometry (*N*) of binding. Top panels show raw data, and bottom panels show fitted curves. Note that it was not possible to fit the data for the non-phosphorylated peptide (n.d.: not determined).

**Figure 4:**
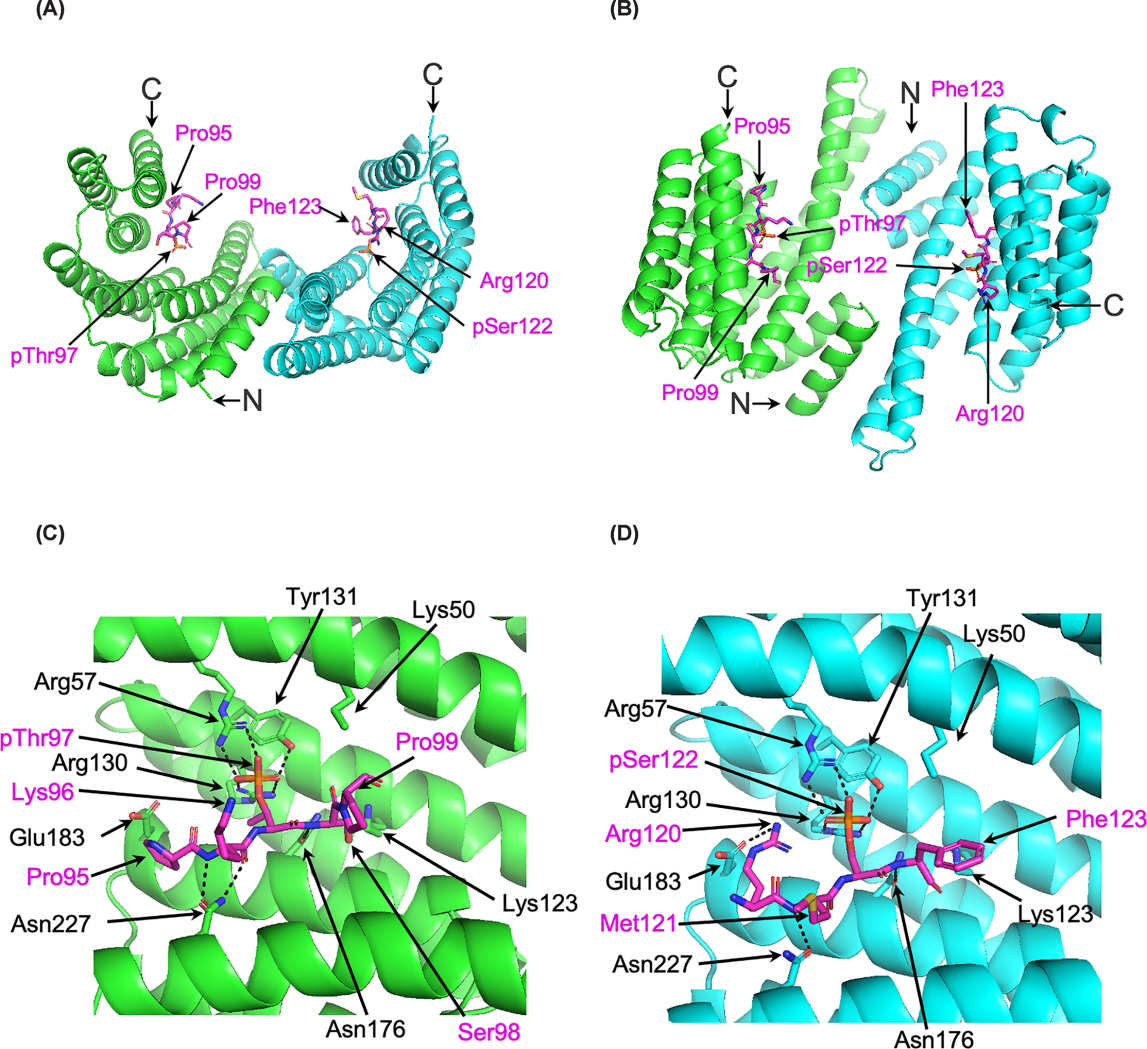
Crystal structure of 14-3-3ε/γ2c-pT97-pS122 complex Top view (**A**) and side view (**B**) of the crystal structure of 14-3-3ε dimer within the γ2 diphosphorylated peptide γ2c-pT97-pS122. Each monomer of 14-3-3ε is coloured in green (site 1) and cyan (site 2). (**C,D**) Two fragments of γ2c-pT97-pS122 peptide occupy 14-3-3ε binding to site 1 and site 2, respectively. The residues involved in hydrogen bond formation are labelled. Backbone of the peptide is shown in magenta.

We measured the binding affinity of synthetic peptides based on the sequence of γ2a spanning T97 and S122 to 14-3-3ε using isothermal titration calorimetry (ITC) (Figure 3). The di-phosphorylated peptide (γ2a-pT97-pS122) exhibited the highest affinity with a dissociation constant (*K*_d_) of 123 nM, approximately 20-fold higher than that of the mono-phosphorylated γ2a-pT97 peptide and >60-fold higher than the mono-phosphorylated γ2a-pS122 peptide. The binding affinity for the di-phosphorylated peptide with 14-3-3ε is consistent with values observed in previous studies [25]. The canonical 14-3-3 binding mechanism relies on phosphorylated serine/threonine residues with flanking residues around the phosphorylation sites contributing to binding [26,27]. To further characterise 14-3-3 binding to the γ2 peptide, we solved the crystal structure of the 14-3-3ε/γ2a-pT97-pS122 peptide co-complex at a resolution of 2.17 Å (crystallographic statistics are presented in Supplementary Table S2; coordinates deposited in the Protein Databank, PDB ID: 29II). In the 3D model, a 14-3-3ε dimer accommodates phosphorylated peptide fragments from the γ2a-pT97-pS122 peptide in each binding groove (Figure 4A). To distinguish between the pThr97 and pSer122 residues within each 14-3-3ε binding groove, the neighbouring residues around each phosphorylated site helped delineate the pThr97 and pSer122 sites against the electron density map. In this structure, Arg57, Arg130, and Tyr131 from each 14-3-3ε monomer contact pThr97 or pSer122 of the peptide, along with several flanking residues (Figure 4B,C). The primary interactions with 14-3-3ε are contributed by pThr97 and pSer122, while the surrounding residues also participate in binding. The 14-3-3ε residues involved in binding are highly conserved across the 14-3-3 family and have been widely reported in interactions with other 14-3-3 binding partners [26,27].

**Figure 5:**
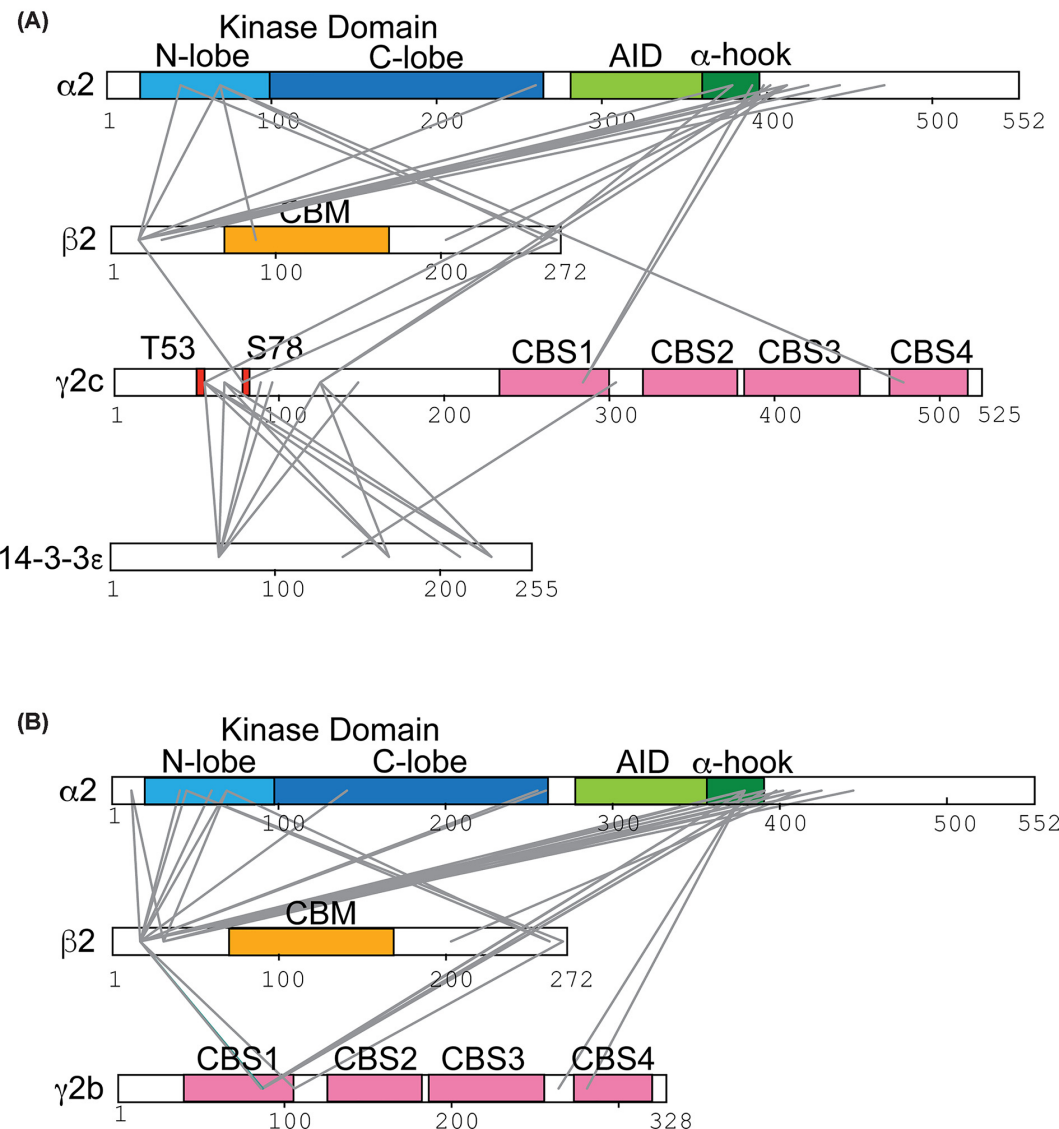
Cross-linking mass spectrometry identifies potential interaction sites in the AMPKγ2c/14-3-3ε complex Purified, recombinant pThr172 AMPKγ2c/14-3-3ε complex (**A**) or pThr172 AMPKγ2b complex (**B**) (in each case 10 μl 7.4 μM) was incubated with 10 μl DSSO (2 mM) for 20 min at 25°C. Cross-linking was quenched by the addition of 500 mM ammonium bicarbonate (0.1 v/v), and 10 μg of treated protein was used for mass spectrometry analysis (as described in 'Materials and methods' section). Functional domains for each of the AMPK subunits are indicated: α2 kinase domain (KD) N- and C-lobes, AID, α-hook region (also known as the α-RIM), carbohydrate binding module, and CBS domains (CBS1–CBS4). The positions of T53 (equivalent to T97 in γ2a) and S78 (equivalent to S122 in γ2a) in γ2c are also shown. Inter-subunit cross-links are shown in grey. No interaction between AMPKγ2b and 14-3-3ε was detected.

We also investigated the interactions between 14-3-3 and AMPK subunits in the AMPKγ2c/14-3-3 co-complex using cross-linking mass spectrometry (Figure 5A and Supplementary Table S3). The data confirmed that the N-terminal extension of γ2c covering T53 and S78 interacts with 14-3-3. Within this region, γ2 also appears to interact with the C-terminal region of α, close to the auto-inhibitory domain (AID) and α-hook region [21], also known as the α-RIM (regulatory subunit-interacting motif) [28]. Additionally, we observed reduced interactions between the N-lobe of the α kinase domain and the N-terminal region of β, in comparison with those seen in the AMPKγ2b complex (Figure 5B and Supplementary Table S4).

### The AMPKγ2c/14-3-3 co-complex has lower activity but is more highly activated by a small molecule AMPK activator compared with AMPKγ1 and AMPKγ2b

**Figure 6:**
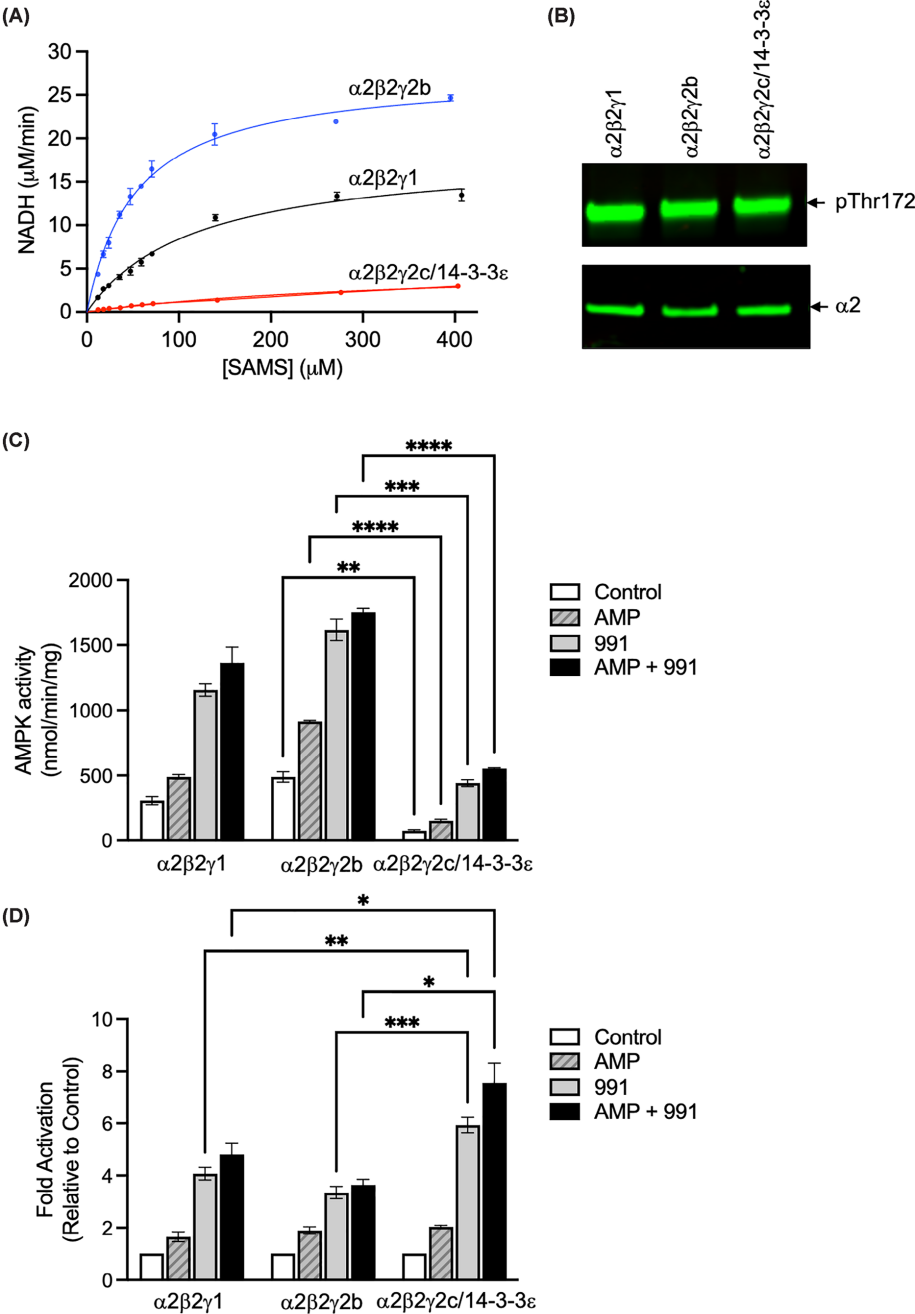
Kinase activity of different AMPK complexes (**A**) AMPK activity of different AMPK complexes was measured using a coupled NADH assay. AMPK complexes (1.5 μM) were titrated with 5 mM SAMS peptide at 20°C. The rate of reaction is shown as the amount of NADH utilised per minute (μM/min), determined by measuring the decrease in absorbance at 340 nm. Experiments were carried out in triplicate, and mean ± SEM are shown. Curve fitting was done in GraphPad Prism using the Michaelis–Menten equation. (**B**) Aliquots of the AMPK complexes used for kinase assay were analysed for phosphorylation of Thr172 by Western blot analysis together with total AMPKα2. (**C**) Allosteric activation of the AMPK complexes by AMP (20 μM) and 991 (10 μM) was determined using the SAMS peptide assay. Results are the mean ± SEM (*n* = 4 independent experiments), and AMPK activity is shown as nmol phosphate incorporated per minute per mg AMPK. Statistical significance was assessed using two-way ANOVA. There was a significant reduction in activity of the α2β2γ2c/14-3-3 complex compared with either α2β2γ1 or α2β2γ2b under all conditions tested, but for clarity, only significant differences between α2β2γ2b and α2β2γ2c/14-3-3 complexes are shown: **P <0.01, ***P <0.001, ****P <0.0001. (**D**) Data from panel (C) was re-plotted to show the fold-activation relative to AMPK activity measured in the absence of AMP and 991. Statistical significance was assessed using two-way ANOVA, and significant differences between α2β2γ2c/14-3-3 and either α2β2γ1 or α2β2γ2b are indicated: *P <0.05, **P <0.01, ***P <0.001.

We determined the activity of recombinant AMPKγ2c/14-3-3ε co-complex using the SAMS peptide as a substrate (Figure 6A). In comparison wit recombinant α2β2γ1 or α2β2γ2b complexes, the AMPKγ2c/14-3-3ε co-complex showed a markedly reduced activity, despite a similar level of Thr172 phosphorylation in each of the complexes (Figure 6B). The *V*_max_ of the AMPKγ2c/14-3-3ε co-complex was about five-fold lower than that of the AMPKγ2b complex and about three-fold lower than the AMPKγ1 complex (Table 1). In addition, the *K*_m_ for the SAMS peptide was 383 μM for the AMPKγ2c/14-3-3ε compared with 53 μM for the AMPKγ2b complex and 115 μM for the AMPKγ1 complex. Furthermore, we assessed the allosteric activation of the complexes by AMP, 991, or both in combination. Despite reduced activity, the AMPKγ2c/14-3-3ε co-complex was activated to a greater extent by 991 (5.9 ± 0.3 fold, *n* = 4) compared with either AMPKγ2b (3.3 ± 0.2 fold, *n* = 4) or AMPKγ1 (3.6 ± 0.35 fold, *n* = 4) complexes (Figure 6C,D).

**Table 1:** Kinetic constants for the α2β2γ2c/14-3-3ε co-complex

Data set	*V*_max_ (μM min^−1^)	*K*_m_ (μM)	*K*_cat_ (min^−1^)	*K*_cat_/*K*_m_
α2β2γ2c/14-3-3ε	5.641 ± 0.591	383.0 ± 62.140	4.49	0.0117
α2β2γ2b	27.57 ± 0.700	53.09 ± 4.132	17.84	0.336
α2β2γ1	18.02 ± 0.856	115.1 ± 11.028	11.38	0.099

The kinetic constants were derived in GraphPad Prism using the Michaelis–Menten equation (*n* = 3 independent replicates).

### AMPKγ2a forms a complex with 14-3-3 in mammalian cells

**Figure 7:**
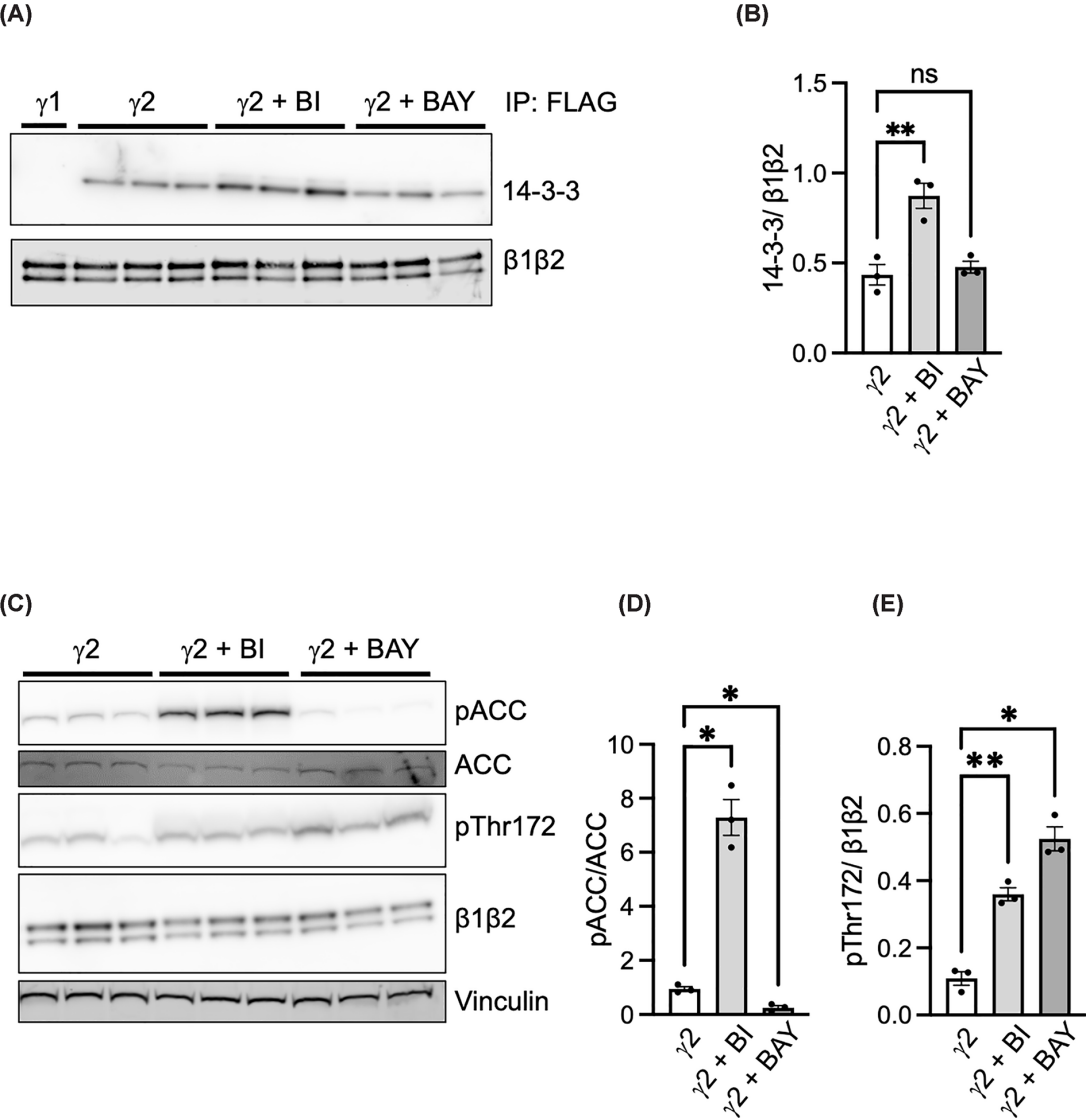
AMPKγ2a expression in HEK293T cells HEK293T cells were transiently transfected with cDNA encoding AMPKγ2a (harbouring an N-terminal FLAG tag). Cells were incubated with either BI9774 (10 μM in 0.1% DMSO), BAY3827 (5 μM in 0.1% DMSO), or 0.1% DMSO alone for 2 h prior to lysis, and in each case 3 independent replicates were performed. (**A**) Complexes were purified using anti-FLAG magnetic beads and analysed by western blotting with antibodies against AMPKβ1/β2 or 14-3-3. The immune complex from cells transfected with AMPKγ1 (harbouring an N-terminal FLAG tag) is shown for comparison. (**B**) Quantification of 14-3-3 relative to AMPKβ1/β2 for the AMPKγ2a complex. In parallel, lysates from cells transfected with AMPKγ2a were analysed by western blotting with antibodies against pACC, total ACC, or pThr172 or AMPKβ1/β2 (**C**). Vinculin was used to monitor total protein expression. Quantification of pACC to total ACC (**D**) and pThr172/AMPKβ1/β2 (**E**). Results shown are mean ± SEM (*n* = 3). Statistically significant differences between non-treated cells and cells treated with either BI9774 or BAY3827 were determined by Student’s *t*-test and are indicated: *P <0.05, **P <0.01; ns denotes no significant difference.

Given our findings using bacterially expressed recombinant AMPK, we sought to determine whether the long forms of γ2 interact with 14-3-3 in mammalian cells. As shown in Figure 7, 14-3-3 forms a stable complex with AMPKγ2a-containing complexes isolated by immunoprecipitation. There was a significant increase in 14-3-3 binding following treatment of the cells with the AMPK activator, BI9774. In contrast, there was no difference in binding following treatment of the cells with the AMPK inhibitor, BAY3827, despite a significant reduction in the phosphorylation of ACC. These findings indicate that while autophosphorylation of AMPK might increase 14-3-3 binding, it does not appear to be essential. This could indicate that other protein kinases phosphorylate γ2a/c to promote 14-3-3 binding.

## Discussion

Despite significant effort, the biological relevance of the different AMPK γ isoforms remains largely enigmatic. Gene expression data shows that there are marked cell- and tissue-dependent differences in mRNA levels of the γ isoforms, suggesting that the isoforms may have cell-specific roles [1]. Another obvious difference is that γ3, and variants of γ2, contain N-terminal extensions that are not present in the γ1 isoform. This raises the possibility that the variable regions within γ2 and γ3 could target AMPK complexes to different subcellular locations and/or different substrates within the cell. In the present study we adopted a biochemical approach to investigate differences between the long forms of γ2 (γ2a and γ2c) and the short form (γ2b) to help address this issue. A key finding from our study is that γ2a and γ2c AMPK complexes form a stable co-complex with 14-3-3. Moreover, the formation of the AMPK-14-3-3 co-complex itself appears to be required for the stabilisation of recombinant AMPKγ2a/γ2c in bacteria. The N-terminal extension in γ2a/γ2c is predicted to be intrinsically disordered, which could account for the difficulty in expressing a stable AMPK complex in the absence of other factors. Importantly, we show that AMPKγ2a forms a stable complex with 14-3-3 in mammalian cells, strengthening the physiological significance of this interaction. Our results also suggest that in mammalian cells the interaction with 14-3-3 can occur in the absence of AMPK activity, indicating that other kinases are capable of phosphorylating the N-terminus of γ2a/c to promote 14-3-3 binding.

Closer inspection of the protein sequence of the γ2 N-terminal extension identified 3 phosphorylation sites (T97, S122, and S131, numbering according to γ2a) that are highly scored as 14-3-3 binding sites. Previous studies have shown that 14-3-3 can bind to phosphorylation sites within regions of high intrinsic disorder and that 14-3-3 binding could favour a transition from disorder to order [29]. By co-expressing AMPKα2β2γ2c and 14-3-3, we were able to obtain a stable, highly purified co-complex between AMPKγ2c and 14-3-3. Formation of the co-complex, however, required phosphorylation of Thr172 within the activation loop of the α subunit. The most likely explanation for this requirement is that 14-3-3 binding almost always involves binding to phosphorylated residues within the target protein [24,29,30] and that AMPK activity is necessary for phosphorylation of the γ2 N-terminal extension in the recombinant bacterial system. Supporting this hypothesis, we identified phosphorylation of T53 and S78 in γ2c (equivalent to T97 and S122 in γ2a) in the purified AMPKγ2c/14-3-3 co-complex. These sites are likely to be autophosphorylation sites, as AMPK, but not CAMKK2, was able to phosphorylate a synthetic peptide spanning T97 and S122 *in vitro*. Further supporting the involvement of these phosphorylation sites in the binding of 14-3-3, we showed that 14-3-3 binds weakly to peptides containing either phosphorylated T97 or S122 and strongly to the diphosphorylated peptide. Furthermore, we obtained the crystal structure of 14-3-3 bound to the doubly phosphorylated peptide. Taken together, these findings are consistent with a model in which AMPK autophosphorylates T97 and S122 within the intrinsically disordered N-terminal extension of γ2 promoting binding of 14-3-3 and stabilisation of the AMPK complex.

We have not been able to purify AMPKγ2a or AMPKγ2c in the absence of 14-3-3, so we cannot compare directly the 14-3-3 bound and unbound forms. However, relative to either AMPKγ1 or AMPKγ2b, AMPKγ2c/14-3-3 exhibits a markedly lower catalytic activity (∼5-fold lower *V*max compared with AMPKγ2b) using the SAMS peptide as a substrate. A clue to the reason for the decreased activity of the AMPKγ2c/14-3-3 complex comes from the cross-linking studies (Figure 5), where there was evidence for interactions between Thr97 and Ser122 with both 14-3-3 and the AID and α-hook regions within the α subunit. The α-hook region plays a key role in communicating the nucleotide status within CBS3 and the subsequent activation state of AMPK [21,28]. The AID region is thought to act as a conformational switch, either binding to the kinase domain to inhibit AMPK activity or being released, depending on which AXP is bound within CBS3 [21,28,31,32]. If the N-terminal region of the long forms of γ2 also interacts with the AID and α-hook/α-RIM regions, it is plausible that this could impact the mechanism regulating kinase activity. Despite extensive efforts, we have been unable to obtain detailed structural information for the AMPKγ2c/14-3-3 complex, so the mechanism for the reduced activity remains to be determined.

To our knowledge, there is very limited data regarding the protein expression of the different γ2 variants in cells or tissues and no studies examining the activity of the variants. A barrier to carrying out such studies is the requirement for antibodies that distinguish between the different γ2 variants. Although two previous studies used antibodies that were reported to be specific for either γ2-3B [16] or the long forms of γ2 [17], data supporting their specificity was lacking, and in the case of the γ2 long form antibody, details of how the antibody was generated were not given. Determining the expression pattern and activity of the different γ2 variants raises technical challenges given that the γ2a variant encompasses both the γ2b and γ2c sequences. Distinguishing the variants by western blotting following separation by SDS–PAGE might be possible, but determining their activity would require steps to sequentially remove different variants by exhaustive immunoprecipitation using selective antibodies. As far as we are aware, this has not been reported. Our findings, however, raise the intriguing possibility that AMPK complexes containing either γ2a or γ2c could be subject to a new mode of regulation involving 14-3-3 binding. While we showed that T97 and S122 can be autophosphorylated in the bacterial expression, our findings in HEK293T cells indicate that the sites required for 14-3-3 binding can be phosphorylated by other kinases, since we did not detect any difference in binding when the cells were treated with the AMPK inhibitor, BAY3827 (Figure 7). Moreover, it is possible that there could be additional phosphorylation sites within the N-terminal extension that could impact T97 and/or S122 phosphorylation or have a direct effect on 14-3-3 binding. Relevant to this point, a previous study reported that ULK1 phosphorylates γ2 on S124 [33]. In the same study, it was also reported that S124 might be an autophosphorylation site, although we have not detected phosphorylation of S124 in our studies using bacterially expressed AMPK. However, given the close proximity of these sites, it is possible that phosphorylation of one site affects phosphorylation of the other. If this were the case, it would raise the possibility that ULK1 phosphorylation of S124 could affect phosphorylation of S122 and potentially alter 14-3-3 binding. This additional level of regulation could provide different signalling pathways to regulate AMPK γ2 function. A recent study identified 4 phosphorylation sites within the N-terminal extension of γ2a (S113, S143, S162, and S196) following expression of recombinant AMPK complexes in HEK293 cells but did not report phosphorylation of T97 or S122 [34]. Taken together, our studies reveal another tier of regulation of AMPKγ2 complexes and warrant further studies exploring the phosphorylation of the N-terminal extension of γ2 and the effect on 14-3-3 binding of the regulation of AMPK in mammalian cells.

## Materials and methods

### Proteins and peptides

All peptides used in this study were synthesised by the Chemical Biology Science Technology Platform (Francis Crick Institute). Novex™ Sharp Pre-stained Protein Standard (Thermo Fisher: LC5800) was used as a marker for SDS–PAGE.

### Recombinant protein expression and purification from bacteria

Recombinant AMPKα2β2γ1, α2β2γ2b, CAMKK2, and 14-3-3ε (harbouring an N-terminal hexahistidine tag cloned in pET-3d) were purified as previously described [35]. Initial attempts to express AMPKγ2a/c complexes in bacteria utilised a tricistronic vector (in pET28a) harbouring α2 (either with an N-terminal hexahistidine tag or no tag), β2 (no tag) and γ2a/c (either with an N-terminal hexahistidine tag, a Twin Strep-tag or no tag). Plasmids were transformed into a range of chemically competent bacteria and selected by growth on agar plates containing 100 μg/ml kanamycin. Single colonies were used to inoculate 1 litre of either LB (Luria Broth) or TB (Terrific Broth) media containing 100 μg/ml kanamycin and cells grown at 30°C. A range of different conditions were tested to improve yield, solubility and stability of the complexes and are summarised in Supplementary Table S1).

For purification of AMPKα2β2γ2a/c-14-3-3 co-complexes, One Shot BL21 Star (DE3) chemically competent bacteria (Thermo Fisher: C601003) were transformed with a tricistronic plasmid (pET28a) harbouring α2 (no tag), β2 (no tag) and γ2a/c (with an N-terminal Twin Strep-tag) together with 14-3-3ε (harbouring an N-terminal hexahistidine tag cloned in pET-3d), and colonies were selected by growth on agar plates containing 100 μg/ml kanamycin and 100 μg/ml ampicillin. A single colony was used to inoculate 1-6 litres of LB medium containing 100 μg/ml kanamycin and 100 μg/ml ampicillin. Cells were grown at 37°C until the OD_600_ reached between 0.6 and 0.8, and protein expression was induced by the addition of isopropyl β-d-1-thiogalactopyranoside (0.1 mM final), and cells were incubated for a further 64 h at 18°C. Bacterial lysate was loaded onto a His-Trap column (GE Healthcare; 17-5248-02), and bound protein was eluted with a linear gradient of 20 to 400 mM imidazole in 300 mM NaCl; 50 mM Tris; 0.5 mM Tris(2-carboxyethyl)phosphine (TCEP), pH 8.0 (buffer A). In some cases, the eluates were incubated at 18°C overnight in the presence of recombinant CAMKK2, 0.5 mM ATP, and 2.5 mM MgCl_2_. The eluate was loaded onto a StrepTrap column (GE Healthcare; 28-9075-47) and washed with 10 column volumes of buffer A, and bound protein was eluted using buffer A containing 3 mM d-desthiobiotin (Sigma–Aldrich; D1411). Eluted protein was applied to a HiLoad Superdex 200pg 16/600 gel-filtration column, and fractions were used for subsequent analysis.

### Western blot analysis

Proteins were resolved by SDS–PAGE on a 12% Bis-Tris gel (Invitrogen NuPAGE; NP0322PK2) and transferred to a polyvinylidene difluoride membrane (Thermo Fisher; 88518). Membranes were probed with primary antibodies at 1:2000 dilution. The following antibodies were from Cell Signalling: mouse anti-α1/2 (#2793), rabbit anti-pan 14-3-3 (#8312), rabbit anti-α1 (#2795), rabbit anti-α2 (#2757), rabbit anti-γ1 (#4187), rabbit anti-His (#2365), rabbit anti-pThr172 (#2535), rabbit anti-pACC (#3661), and rabbit anti-ACC (#3662). Mouse anti-FLAG M2 (Sigma–Aldrich; F3165), mouse anti-His (Cell Biolabs; AKR-003), rabbit anti-β2 (Thermo Fisher; PA5-112885), rabbit anti-γ1 (Proteintech Europe; 10290-1-AP20), and rabbit anti-γ2 C-terminus (GeneTex; GTX114178S) were also used. After extensive washing, membranes were incubated for 1 h with secondary antibodies (IRDye 680RD donkey anti-mouse IgG; IRDye 800CW donkey anti-rabbit IgG; LI-COR Biosciences) diluted 1:10,000. Blots were imaged on a LI-COR Odyssey CLx or GE Amersham Imager 680. All steps were carried out at room temperature.

### Size exclusive chromatography coupled to multi-angle light scattering

Proteins were dialysed overnight at 4°C against a buffer containing 150 mM NaCl, 50 mM HEPES, 3 mM sodium azide, and 0.5 mM TCEP (pH 8.0). Following dialysis, protein was concentrated to 2.4 mg/ml, filtered using a 0.22-μm tube filter (Corning; 10104101), and 100 μl loaded onto a Superdex 200 10/300 GL column (Sigma–Aldrich; GE17-5175-01) connected to a Wyatt MALS System. The scattered light intensity was monitored using an in-line DAWN-HELEOS II laser photometer, and the concentration of fractionated protein was recorded using an OPTILAB-TrEx differential refractometer. All measurements were performed at room temperature.

### Phosphorylation site identification by mass spectrometry

Recombinant AMPKα2β2γ2c/14-3-3 protein isolated from *E. coli* was processed using an in-solution digestion procedure as previously described [9]. Data were processed using the MaxQuant software platform (v1.6.2.3) [36], with database searches carried out by the built-in Andromeda search engine against the UniProt Human database (version 20180104, number of entries: 161,549) and *E. coli* database (version 20180723, number of entries: 4497). A reverse decoy database approach was used at a 1% false discovery rate for peptide spectrum matches and protein identification. Search parameters included: maximum missed cleavages set to 2, fixed modification of cysteine carbamidomethylation, and variable modifications of methionine oxidation and protein N-terminal acetylation and phosphorylation (STY). A combination of the MaxQuant visualizer and Thermo Freestyle software was used to inspect fragmentation spectra and XIC traces for phosphosites of interest.

### Native mass spectrometry

Forty microliters of 2 mg/ml protein was exchanged into 200 mM ammonium acetate buffer using a Micro Bio-Spin 6 column (Bio-Rad). The sample was introduced by static nanospray (nano ESI capillaries) and acquired on a Thermo Ultra-High Mass Resolution (UHMR) mass spectrometer. The Xcalibur program (Thermo Scientific) was used for data analysis.

### Cross-linking mass spectrometry

One milligram of DSSO (disuccinimidyl sulfoxide, Thermo Fisher; A33545) was dissolved in 10.3 μl anhydrous DMSO (dimethyl sulfoxide, Sigma; D2650) to make a 250 mM stock solution, which was diluted to 2 mM in 50 mM HEPES, 300 mM NaCl, and 0.5 mM TCEP (pH 8.0). Purified AMPK (10 μl of a 7.4 μM solution) was incubated with 10 μl DSSO (2 mM) for 20 min at 25°C. The cross-linking reaction was stopped by adding 2 ml ammonium bicarbonate (500 mM ammonium bicarbonate; Sigma: A6141) and incubating for a further 15 min at 37°C. The cross-linked protein (10 μg) was processed using an in-solution digestion with trypsin and analysed by mass spectrometry, carried out by the Proteomics STP, Francis Crick Institute. Cross-link map was generated using an online tool xiNET [37].

### Far-UV circular dichroism

Three hundred microliters of 0.2 mg/ml protein solution in 100 mM NaCl, 25 mM HEPES, and 1 mM TCEP (pH 8.0) was placed in a 1-mm fused silica cuvette. The far-UV CD spectra measurement over 200–260 nm was obtained using a Jasco J-815 spectropolarimeter at 20°C. The secondary structure contents were analysed from far-UV CD spectra using an open-source software CDPro [38].

### Isothermal titration calorimetry

Peptides spanning residues 89–128 of AMPKγ2a were synthesised as follows: non-phosphorylated (γ2a-T97-S122), singly phosphorylated (γ2a-pT97-S122 and γ2a-T97-pS122), and doubly phosphorylated (γ2a-pT97-pS122). Peptides and recombinant 14-3-3ε protein were dialysed against ITC buffer (150 mM NaCl, 20 mM Tris, 0.5 mM TCEP, pH 8.0) overnight at 4°C and filtered using 0.22 μm syringe filters (Millipore; SLGP033N). Peptides were diluted in ITC buffer to 400–600 μM and 14-3-3ε diluted to 20–30 μM. The ITC system (MicroCal iTC200, Malvern) was initialised using ITC buffer in the injection syringe, and the ITC cell equilibrated at 20°C. Peptide was initially injected in a 0.4 μl aliquot (in 0.8 s, stirring speed of 750 rpm) into the ITC cell containing 300 μl of 14-3-3ε and subsequently in 2 μl aliquots (in 4 s). The spacing time between the two injections was 180 s. The raw data from three independent experiments were analysed using the PEAQ-ITC Analysis Software (Malvern).

### AMPK assay

AMPK activity was measured by phosphorylation of the SAMS peptide determined either by incorporation of ^32^P-labelled phosphate from ATP [39] or using a coupled assay. Briefly, for the coupled assay, 15 μl pyruvate kinase/lactate dehydrogenase mix (Sigma–Aldrich; P0294) and 10 μl AMPK protein were added to 400 μl buffer (50 mM HEPES (pH 7.5), 100 mM NaCl, 10 mM MgCl_2_, 0.2 mM ATP, 0.1 mM phosphoenolpyruvate, 0.15 mM NADH) in a quartz micro-cuvette with 10 mm pathlength. The mixture was titrated with increasing concentrations of SAMS peptide, and the absorbance at 340 nm was recorded using a V550 UV/VIS spectrophotometer (JASCO) at 20°C. In this assay, the conversion of ATP to ADP is monitored by the conversion of NADH to NAD^+^ (measured by a change in absorbance at 340 nm) due to the presence of pyruvate kinase and lactate dehydrogenase. The raw data was analysed using GraphPad Prism 8.3 software. The constants of *V*_max_ and *K*_m_ were calculated by non-linear least squares regression on the Michaelis–Menten equation.

### X-ray crystallography

Recombinant 14-3-3ε protein (20 mg/ml) was incubated with the doubly phosphorylated γ2c-pT53-pS78 peptide at a 1:1.1 molar ratio on ice for 1 h, then filtered using a 0.22-μm tube filter. 0.1 μl of the sample was mixed with either 0.1 μl or 0.15 μl of well reservoir buffer (20% (w/v) polyethylene glycol 3350, 100 mM BIS-TRIS propane (pH 6.5), 200 mM sodium fluoride). Crystals were mounted in a cryoprotectant prepared by adding 30% PEG400 in the reservoir buffer. Diffraction data were collected at Diamond Light Source (Oxford, U.K.) on beamline I03 and then processed on the beamline using the Xia2 DIALS software with an output in ‘mtz’ file format. The structure was solved using molecular replacement using Phaser software [40]. The structure was built into density using the Coot molecular graphics package [41], and modelling refinement was carried out using the Phenix.refine software [41]. Figures were generated using Pymol.

### Expression of AMPK complexes in mammalian cells

HEK293T cells were transfected with cDNA encoding either AMPKγ1 or AMPKγ2a (both harbouring a FLAG tag at the N-terminus) as previously described [19]. Twenty-four hours after transfection, cells were incubated in the presence or absence of either BI9774 (10 mM) or BAY3827 (5 mM) for 2 h, and AMPK complexes were isolated from cell lysates by immune capture with FLAG M2 magnetic beads (Sigma–Aldrich, M8823). Immune complexes were analysed by western blotting to determine the presence of 14-3-3. In parallel, cell lysates were used to determine ACC and Thr172 phosphorylation.

## Supplementary Material

Supplementary Figure S1-S3 and Tables S1-S4

## Data Availability

Atomic co-ordinates for the structure of 14-3-3ε bound to the γ2a pT97-pS122 peptide are available from the Protein Data Bank (ID: 29II) [42]. All other data are included in the manuscript.

## Open Access

Open access for this article was enabled by the participation of Imperial College London in an all-inclusive *Read & Publish* agreement with Portland Press and the Biochemical Society under an agreement with JSIC.

## Abbreviations

ACC: acetyl-CoA carboxylase
AID: auto-inhibitory domain
AMPK: AMP-activated protein kinase
CAMKK2: calcium/calmodulin dependent protein kinase kinase 2
CD: circular dichroism
CBS: cystathionine-β-synthase
DMSO: dimethyl sulfoxide
DSSO: disuccinimidyl sulfoxide
HEPES: 4-(2-hydroxyethyl)-1-piperazineethanesulfonic acid
ITC: isothermal titration calorimetry
mAU: milli-absorbance units
SAMS: the synthetic peptide HMRSAMSGLHLVKRR
SEC: size exclusion chromatography
SEC-MALS: size exclusion chromatography-multi-angle light scattering
Thr172: threonine 172
TCEP: Tris(2-carboxyethyl)phosphine
