# Supplementary material for: Binding of 14-3-3 stabilises recombinant AMPKγ2-containing complexes
Source: Biochem J. 2026 Apr 13;483(5):621–37. doi: 10.1042/BCJ20250342 (PMC13142926; doi:10.1042/BCJ20250342)
Supplement: Supplementary Figure S1-S3 and Tables S1-S4 [file BCJ-2025-0342_supp.pdf]

Supplementary Table 1. Summary of different conditions used for expression of AMPKα2β2γ2a/c complexes in the absence of 14-3-3 co-expression.

Variable	Condition	Impact
Tag	Hexahistidine, Twin-Strep, GST	No obvious effect
Tag position	N- vs C-terminal	No obvious effect
Bacterial strain	BL21, BL21 star, BL21 pLysS, Rosseta	No obvious effect
Culture medium	LB vs TB	No obvious effect
Induction time (OD_{600})	Range: 0.5-1	No obvious effect
Induction temperature	Range: 16-37 °C	No obvious effect
Induction time	Range: 16-64 hours	No obvious effect
Purification buffer	NaCl concentration<br>Range: 0.3-1.5M<br>pH Range: 5-9<br>Additives: glycerol, arginine	Improved solubility >1 M, but poorer stability and subunit stoichiometry<br>No obvious effect<br>No obvious effect

The different conditions used during the study are listed, and where a range of conditions were tested, the upper and lower limits of the range are indicated.

Supplementary Table 2. Data collection and refinement statistics.

	14-3-3ε with γ2a pT97-pS122 peptide bound (PDB ID: 29II)
Data Collection	
X-ray Source	Diamond I03
Wavelength (Å)	0.9763
Space group	*P*21 2 21
Cell dimensions	
a, *b*, *c* (Å)	85.07, 118.18, 65.64
α, β, γ (°)	90, 90, 90
Resolution (outerShell/overall) (Å)	2.17-2.23/2.17-69.04
$R_{merge\ (\%)}$	3.424
I/σ(I)	1.1
CC(1/2)	0.7
Completeness (%)	99.8
Redundancy	11.2
Refinement	
Resolution (Å)	2.17
No. reflections (total)	446425
No. reflections (unique)	35743
R_{work}/R_{free}	0.235/0.292
No. atoms	7446
Protein	7068
Ligand	154
Water	75
r.m.s deviations	
Bond lengths (Å)	0.0159
Bond angles (°)	2.96
Ramachandran favored (%)	94.57
Ramachandran outliers (%)	0.87

Supplementary Table 3. Peptides identified in crosslinking mass spectrometry with the AMPKγ2c/14-3-3 complex.

Protein1	Peptide1 Sequence	Link Pos1	Protein2	Peptide2 sequence	Link Pos2
14-3-3ε	NLLSVAYKNVIGAR	8	AMPKγ2c	GPQPRPSSPMSAPVRPKTSPGSPKTVF PFSYQESPPR	24
14-3-3ε	VFYYKMK	5	14-3-3ε	NLLSVAYKNVIGAR	8
14-3-3ε	NLLSVAYKNVIGAR	8	AMPKγ2c	KVDSPFGPGSPSKGFFSR	13
14-3-3ε	NLLSVAYKNVIGAR	8	AMPKγ2c	SSSKESSPNSNPATSPGGIR	4
14-3-3ε	HLIPAANTGESKVFYYK	12	14-3-3ε	MKGDYHR	2
14-3-3ε	NLLSVAYKNVIGAR	8	AMPKγ2c	KVDSPFGPGSPSKGFFSR	1
14-3-3ε	NLLSVAYKNVIGAR	8	14-3-3ε	AAFDDAIAELDTLSEESYKDSTLIMQLLR	19
14-3-3ε	KVAGMDVELTVEER	1	14-3-3ε	LICCDILDVLDKHLIPAANTGESK	12
14-3-3ε	MKGDYHR	2	AMPKγ2c	KQSFVGMLTITDFINILHRYYK	1
14-3-3ε	MKGDYHR	2	14-3-3ε	LICCDILDVLDKHLIPAANTGESK	12
14-3-3ε	EAAENSLVAYKAASDIAMTELPPTHPIR	11	AMPKγ2c	SSSKESSPNSNPATSPGGIR	4
14-3-3ε	NLLSVAYKNVIGAR	8	AMPKγ2c	GPQPRPSSPMSAPVRPKTSPGSPK	17
AMPKα2	EIQNLKLFR	6	AMPKα2	SLDVVGKIK	7
AMPKα2	MPPLIADSPKAR	10	AMPKα2	IMNQASEFYLASSPPSGSFMDDSAMHIP PGLKPHPER	32
AMPKα2	EIQNLKLFR	6	AMPKα2	MPPLIADSPKAR	10
AMPKα2	CPLDALNTTKPKSLAVK	12	AMPKα2	MPPLIADSPKAR	10
AMPKα2	MPPLIADSPKAR	10	AMPKα2	CPLDALNTTKPK	10
AMPKα2	ATIKDIR	4	AMPKα2	EHEWFKQDLPSYLFPEDPSYDANVIDDE AVKEVCEK	6
AMPKα2	DLKPENVLLDAHMNAK	3	AMPKα2	VAVKILNR	4
AMPKα2	MPPLIADSPKAR	10	AMPKβ2	KKYVTTLLYKPI	1
AMPKα2	MPPLIADSPKAR	10	AMPKα2	SYLLDFKSIDDEVVEQR	7
AMPKα2	MPPLIADSPKAR	10	AMPKα2	SQSKPYDIMAEVYR	4
AMPKα2	SQSKPYDIMAEVYR	4	AMPKα2	ATIKDIR	4
AMPKα2	VAVKILNR	4	AMPKα2	SLDVVGKIKR	7
AMPKα2	SLAVKK	5	AMPKα2	MPPLIADSPKAR	10
AMPKα2	SQSKPYDIMAEVYR	4	AMPKα2	ATIKDIR	4
AMPKα2	CPLDALNTTKPKSLAVK	12	AMPKα2	AKWHLGIR	2
AMPKα2	VAVKILNR	4	AMPKβ2	KYVTTLLYKPI	9
AMPKα2	SQSKPYDIMAEVYR	4	AMPKβ2	HGAKAAR	4
AMPKα2	SQSKPYDIMAEVYR	4	AMPKγ2c	SSSKESSPNSNPATSPGGIR	4
AMPKα2	AKWHLGIR	2	AMPKα2	SLAVKK	5
AMPKα2	VKIGEHQLTGHK	2	AMPKα2	VAVKILNR	4

AMPKα2	AMKQLDFEWK	3	AMPKβ2	SEGAGGHAPGKEHK	11
AMPKα2	MPPLIADSPKAR	10	AMPKβ2	HGAKAAR	4
AMPKα2	SVATLLMHMLQVDPLKR	16	AMPKα2	ATIKDIR	4
AMPKα2	SQSKPYDIMAEVYR	4	AMPKα2	EIQNLKLFR	6
AMPKα2	IMNQASEFYLASSPPSGSFMDDSAMHI PPGLKPHPER	32	AMPKα2	MPPLIADSPKAR	10
AMPKα2	SLDVVGKIKR	7	AMPKα2	EIQNLKLFR	6
AMPKα2	CPLDALNTTKPK	10	AMPKγ2c	KAFFALVANGVR	1
AMPKα2	MPPLIADSPKAR	10	AMPKα2	AKWHLGIR	2
AMPKα2	AMKQLDFEWK	3	AMPKβ2	HGAKAAR	4
AMPKα2	EIQNLKLFR	6	AMPKα2	MPPLIADSPKAR	10
AMPKα2	SQSKPYDIMAEVYR	4	AMPKα2	EIQNLKLFR	6
AMPKα2	SYLLDFKSIDDEVVEQR	7	AMPKα2	MPPLIADSPKAR	10
AMPKα2	EIQNLKLFR	6	AMPKβ2	WSEGGKEVFISGSFNNWSTKIPLIK	6
AMPKα2	ATIKDIR	4	AMPKα2	EHEWFKQDLPSYLFPEDPSYDANVIDDE AVK	6
AMPKα2	MPPLIADSPKAR	10	AMPKβ2	HGAKAAR	4
AMPKα2	SQSKPYDIMAEVYR	4	AMPKβ2	FKSPPILPPHLLQVILNK	2
AMPKα2	CPLDALNTTKPK	10	AMPKα2	MPPLIADSPKAR	10
AMPKα2	VAVKILNR	4	AMPKα2	DLKPENVLLDAHMNAK	3
AMPKα2	QKHDGR	2	AMPKα2	VKIGEHQLTGHK	2
AMPKα2	EIQNLKLFR	6	AMPKβ2	KYVTTLLYKPI	1
AMPKα2	CPLDALNTTKPK	10	AMPKα2	SLAVKK	5
AMPKα2	SQSKPYDIMAEVYR	4	AMPKβ2	HGAKAAR	4
AMPKα2	ATIKDIR	4	AMPKα2	MPPLIADSPKAR	10
AMPKα2	SLDVVGKIK	7	AMPKα2	VAVKILNR	4
AMPKα2	VAVKILNR	4	AMPKα2	MPPLIADSPKAR	10
AMPKα2	AMKQLDFEWK	3	AMPKα2	KNPVTGNYVK	1
AMPKα2	VKIGEHQLTGHK	2	AMPKα2	QKHDGR	2
AMPKα2	QKHDGR	2	AMPKα2	IGHYVLGDTLGVGTFGKVK	17
AMPKα2	QKHDGR	2	AMPKα2	VKIGHYVLGDTLGVGTFGK	2
AMPKα2	AKWHLGIR	2	AMPKα2	CPLDALNTTKPK	10
AMPKα2	VAVKILNR	4	AMPKα2	HMVVHRDLKPENVLLDAHMNAK	9
AMPKα2	ATIKDIR	4	AMPKα2	EHEWFKQDLPSYLFPEDPSYDANVIDDE AVK	6
AMPKα2	EIQNLKLFR	6	AMPKα2	IMNQASEFYLASSPPSGSFMDDSAMHIP PGLKPHPER	32
AMPKα2	IMNQASEFYLASSPPSGSFMDDSAMHI PPGLKPHPER	32	AMPKα2	MPPLIADSPKAR	10
AMPKα2	AKWHLGIR	2	AMPKγ2c	SSSKESSPNSNPATSPGGIR	4
AMPKα2	KAKWHLGIR	3	AMPKα2	CPLDALNTTKPK	10

AMPKα2	EIQNLKLFR	6	AMPKα2	SLDVVGKIKR	7
AMPKα2	SYLLDFKSIDDEVVEQR	7	AMPKβ2	HGAKAAR	4
AMPKα2	EIQNLKLFR	6	AMPKβ2	KYVTTLLYKPI	1
AMPKα2	EIQNLKLFR	6	AMPKγ2c	FDVINLAAEKTYNNLDITVTQALQHR	10
AMPKβ2	SEGAGGHAPGKEHK	11	AMPKβ2	HGAKAAR	4
AMPKβ2	HGAKAAR	4	AMPKα2	SYLLDFKSIDDEVVEQR	7
AMPKβ2	HGAKAAR	4	AMPKα2	ATIKDIR	4
AMPKβ2	HGAKAAR	4	AMPKα2	VAVKILNR	4
AMPKβ2	HGAKAAR	4	AMPKγ2c	AAPLWESKK	8
AMPKβ2	HGAKAAR	4	AMPKα2	KNPVTGNYVK	1
AMPKβ2	HGAKAAR	4	AMPKα2	AMKQLDFEWK	3
AMPKβ2	HGAKAAR	4	AMPKα2	EIQNLKLFR	6
AMPKβ2	HGAKAAR	4	AMPKβ2	EVFISGSFNNWSTKIPLIK	14
AMPKβ2	KKYVTTLLYKPI	1	AMPKα2	MPPLIADSPKAR	10
AMPKβ2	HGAKAAR	4	AMPKβ2	WSEGGKEVFISGSFNNWSTKIPLIK	20
AMPKβ2	FKSPPILPPHLLQVILNK	2	AMPKα2	SQSKPYDIMAEVYR	4
AMPKβ2	SEGAGGHAPGKEHK	11	AMPKα2	SQSKPYDIMAEVYR	4
AMPKγ2c	SSSKESSPNSNPATSPGGIR	4	14-3-3ε	NLLSVAYKNVIGAR	8
AMPKγ2c	KVDSPFGPGSPSKGFFSR	1	14-3-3ε	LAKAAFDDAIAELDTLSEESYKDSTLIMQL LR	3
AMPKγ2c	KVDSPFGPGSPSKGFFSR	1	AMPKα2	SQSKPYDIMAEVYR	4
AMPKγ2c	KAFFALVANGVR	1	AMPKγ2c	FDVINLAAEKTYNNLDITVTQALQHR	10
AMPKγ2c	SSSKESSPNSNPATSPGGIR	4	14-3-3ε	AAFDDAIAELDTLSEESYKDSTLIMQLLR	19
AMPKγ2c	SPMVQIYELEEHKIETWR	13	AMPKγ2c	AAPLWESKK	8
AMPKγ2c	AAPLWESKK	8	AMPKβ2	YVTTLLYKPI	8
AMPKγ2c	KVDSPFGPGSPSKGFFSR	1	14-3-3ε	AAFDDAIAELDTLSEESYKDSTLIMQLLR	19
AMPKγ2c	KVDSPFGPGSPSKGFFSR	13	14-3-3ε	AAFDDAIAELDTLSEESYKDSTLIMQLLR	19
AMPKγ2c	KVDSPFGPGSPSKGFFSR	13	14-3-3ε	NLLSVAYKNVIGAR	8
AMPKγ2c	KVDSPFGPGSPSKGFFSR	1	AMPKγ2c	LEVLFQGPMPLLDGDLEGSGKHSSR	21
AMPKγ2c	SSSKESSPNSNPATSPGGIR	4	AMPKγ2c	GPQPRPSSPMSAPVRPKTSPGSPK	17
AMPKγ2c	KVDSPFGPGSPSKGFFSR	1	AMPKγ2c	GPQPRPSSPMSAPVRPKTSPGSPK	17
AMPKγ2c	KAFFALVANGVR	1	AMPKα2	SLAVKK	5
AMPKγ2c	AAPLWESKK	8	AMPKβ2	YVTTLLYKPI	8
AMPKγ2c	KVDSPFGPGSPSKGFFSR	13	14-3-3ε	EAAENSLVAYKAASDIAMTELPPTHPIR	11
AMPKγ2c	SSSKESSPNSNPATSPGGIR	4	14-3-3ε	EAAENSLVAYKAASDIAMTELPPTHPIR	11
AMPKγ2c	KVDSPFGPGSPSKGFFSR	1	14-3-3ε	EAAENSLVAYKAASDIAMTELPPTHPIR	11
AMPKγ2c	KTSGLSSSPSTPTQVTK	1	14-3-3ε	NLLSVAYKNVIGAR	8

AMPKγ2c	SSSKESSPNSNPATSPGGIR	4	AMPKα2	SQSKPYDIMAEVYR	4

Peptides identified from the MS crosslinking experiment shown in Figure 5A are listed. The position of the linked residue in each of the peptides is shown (LinkPos1 and LinkPos2).

Supplementary Table 4. Peptides identified in crosslinking mass spectrometry with the AMPKγ2b complex.

Protein1	PepSeq1	Link Pos1	Protein2	PepSeq2	Link Pos2
AMPKα2	MPPLIADSPKAR	10	AMPKβ2	HGAKAAR	4
AMPKα2	AKWHLGIR	2	AMPKβ2	HGAKAAR	4
AMPKα2	EIQNLKLFR	6	AMPKβ2	HGAKAAR	4
AMPKα2	VKIGEHQLTGHK	2	AMPKα2	VAVKILNR	4
AMPKα2	AKWHLGIR	2	AMPKα2	SLAVKK	5
AMPKα2	MPPLIADSPKAR	10	AMPKα2	VKIGEHQLTGHK	2
AMPKα2	AMKQLDFEWK	3	AMPKβ2	HGAKAAR	4
AMPKα2	CPLDALNTTKPK	10	AMPKα2	SLAVKK	5
AMPKα2	SQSKPYDIMAEVYR	4	AMPKβ2	SEGAGGHAPGKEHK	11
AMPKα2	MPPLIADSPKAR	10	AMPKα2	SLAVKK	5
AMPKα2	SQSKPYDIMAEVYR	4	AMPKβ2	HGAKAAR	4
AMPKα2	AKWHLGIR	2	AMPKα2	ATIKDIR	4
AMPKα2	SLDVVGKIKR	7	AMPKα2	VAVKILNR	4
AMPKα2	MPPLIADSPKAR	10	AMPKα2	ATIKDIR	4
AMPKα2	RKNPVTGNYVK	2	AMPKα2	AMKQLDFEWK	3
AMPKα2	CPLDALNTTKPKSLAVKK	12	AMPKα2	MPPLIADSPKAR	10
AMPKα2	CPLDALNTTKPK	10	AMPKα2	VAVKILNR	4
AMPKα2	MPPLIADSPKAR	10	AMPKα2	SLDVVGKIKR	7
AMPKα2	CPLDALNTTKPKSLAVKK	10	AMPKα2	KAKWHLGIR	3
AMPKα2	VKIGHYVLGDTLGVGTFGK	2	AMPKα2	QKHDGR	2
AMPKα2	MPPLIADSPKAR	10	AMPKα2	VAVKILNR	4
AMPKα2	MPPLIADSPKAR	10	AMPKα2	AKWHLGIR	2
AMPKα2	HMVVHRDLKPENVLLDAHMNAK	9	AMPKα2	VAVKILNR	4
AMPKα2	MPPLIADSPKAR	10	AMPKα2	CPLDALNTTKPK	10
AMPKα2	SLDVVGKIKR	7	AMPKα2	EIQNLKLFR	6
AMPKα2	DLKPENVLLDAHMNAK	3	AMPKα2	SLDVVGKIKR	7
AMPKα2	DLKPENVLLDAHMNAK	3	AMPKα2	SLDVVGKIKR	9
AMPKα2	SQSKPYDIMAEVYR	4	AMPKα2	ATIKDIR	4
AMPKα2	CPLDALNTTKPK	10	AMPKα2	EIQNLKLFR	6
AMPKα2	MPPLIADSPKAR	10	AMPKα2	EIQNLKLFR	6
AMPKα2	SQSKPYDIMAEVYR	4	AMPKα2	MPPLIADSPKAR	10
AMPKα2	SQSKPYDIMAEVYR	4	AMPKα2	EIQNLKLFR	6
AMPKα2	IMNQASEFYLASSPPSGSFMDDSAMHIP PGLKPHPER	32	AMPKα2	MPPLIADSPKAR	10
AMPKα2	SYLLDFKSIDDEVVEQR	7	AMPKα2	MPPLIADSPKAR	10
AMPKα2	EHEWFKQDLPSYLFPEDPSYDANVIDDE AVKEVCEK	6	AMPKα2	ATIKDIR	4
AMPKα2	IGEHQLTGHKVAVK	10	AMPKβ2	HGAKAAR	4
AMPKα2	VAVKILNR	4	AMPKβ2	HGAKAAR	4
AMPKα2	SLDVVGKIKR	7	AMPKβ2	HGAKAAR	4

AMPKα2	CPLDALNTTKPK	10	AMPKβ2	HGAKAAR	4
AMPKα2	DLKPENVLLDAHMNAK	3	AMPKβ2	HGAKAAR	4
AMPKα2	CPLDALNTTKPK	10	AMPKα2	ATIKDIR	4
AMPKα2	EIQNLKLFR	6	AMPKα2	SLAVKK	5
AMPKα2	SQSKPYDIMAEVYR	4	AMPKα2	CPLDALNTTKPK	10
AMPKα2	IGHYVLGDTLGVGTFGKVK	17	AMPKα2	QKHDGR	2
AMPKα2	CPLDALNTTKPKSLAVK	12	AMPKα2	KAKWHLGIR	3
AMPKα2	EIQNLKLFR	6	AMPKα2	AKWHLGIR	2
AMPKα2	MPPLIADSPKAR	10	AMPKγ2b	KAFFALVANGVR	1
AMPKα2	SVATLLMHMLQVDPLKR	16	AMPKβ2	HGAKAAR	4
AMPKα2	VKIGHYVLGDTLGVGTFGK	2	AMPKα2	EIQNLKLFR	6
AMPKα2	IMNQASEFYLASSPPSGSFMDDSAMHIP PGLKPHPER	32	AMPKα2	SQSKPYDIMAEVYR	4
AMPKα2	SVATLLMHMLQVDPLKR	16	AMPKα2	ATIKDIR	4
AMPKα2	SVATLLMHMLQVDPLKR	16	AMPKα2	CPLDALNTTKPK	10
AMPKα2	SVATLLMHMLQVDPLKR	16	AMPKα2	MPPLIADSPKAR	10
AMPKα2	SVATLLMHMLQVDPLKR	16	AMPKα2	SQSKPYDIMAEVYR	4
AMPKα2	VAVKILNR	4	AMPKα2	SLAVKK	5
AMPKα2	IGHYVLGDTLGVGTFGKVK	17	AMPKα2	QKHDGR	2
AMPKα2	CPLDALNTTKPKSLAVK	12	AMPKα2	KAKWHLGIR	3
AMPKα2	EIQNLKLFR	6	AMPKα2	AKWHLGIR	2
AMPKα2	MPPLIADSPKAR	10	AMPKγ2b	KAFFALVANGVR	1
AMPKα2	SVATLLMHMLQVDPLKR	16	AMPKβ2	HGAKAAR	4
AMPKα2	VKIGHYVLGDTLGVGTFGK	2	AMPKα2	EIQNLKLFR	6
AMPKα2	IMNQASEFYLASSPPSGSFMDDSAMHIP PGLKPHPER	32	AMPKα2	SQSKPYDIMAEVYR	4
AMPKα2	SVATLLMHMLQVDPLKR	16	AMPKα2	ATIKDIR	4
AMPKα2	SVATLLMHMLQVDPLKR	16	AMPKα2	CPLDALNTTKPK	10
AMPKα2	SVATLLMHMLQVDPLKR	16	AMPKα2	MPPLIADSPKAR	10
AMPKα2	SVATLLMHMLQVDPLKR	16	AMPKα2	SQSKPYDIMAEVYR	4
AMPKα2	VAVKILNR	4	AMPKα2	SLAVKK	5
AMPKα2	SYLLDFKSIDDEVVEQR	7	AMPKα2	EIQNLKLFR	6
AMPKα2	RKNPVTGNYVK	2	AMPKβ2	HGAKAAR	4
AMPKα2	SQSKPYDIMAEVYR	4	AMPKα2	QKHDGR	2
AMPKβ2	FKSPPILPPHLLQVILNK	2	AMPKα2	SQSKPYDIMAEVYR	4
AMPKβ2	KYVTTLLYKPI	1	AMPKα2	VAVKILNR	4
AMPKβ2	KYVTTLLYKPI	1	AMPKα2	EIQNLKLFR	6
AMPKβ2	KYVTTLLYKPI	9	AMPKα2	VAVKILNR	4
AMPKβ2	SEGAGGHAPGKEHK	11	AMPKα2	MPPLIADSPKAR	10
AMPKγ2b	LVVFDTTLQVKK	11	AMPKα2	SLAVKK	5
AMPKγ2b	LVVFDTTLQVKK	11	AMPKα2	MPPLIADSPKAR	10
AMPKγ2b	KAFFALVANGVR	1	AMPKα2	SLAVKK	5
AMPKγ2b	ISALPVVDESGKVVDIYSK	12	AMPKα2	MPPLIADSPKAR	10
AMPKγ2b	CNKLEILETIVDR	3	AMPKγ2b	QKETETE	2

AMPKγ2b	FDVINLAAEKTYNNLDITVTQALQHR	10	AMPKα2	MPPLIADSPKAR	10
AMPKγ2b	FDVINLAAEKTYNNLDITVTQALQHR	10	AMPKγ2b	LVVFDTTLQVKK	11
AMPKγ2b	LVVFDTTLQVKK	11	AMPKβ2	HGAKAAR	4
AMPKγ2b	KAFFALVANGVR	1	AMPKα2	CPLDALNTTKPK	10
AMPKγ2b	KAFFALVANGVR	1	AMPKβ2	HGAKAAR	4
AMPKγ2b	AAPLWESKK	8	AMPKβ2	HGAKAAR	4
AMPKγ2b	SPMVQIYELEEHKIETWR	13	AMPKγ2b	AAPLWESKK	8

Peptides identified from the MS crosslinking experiments shown in Figure 5B are listed. The position of the linked residue in each of the peptides is shown (LinkPos1 and LinkPos2)

Supplementary Figure S1

A

γ2a MGSAVMDTKKKKDVSSPGGSGGKKNASQKRRSLRVHIPDLSSFAMPLLDGDLEGSGKHSS
γ2c MPLLDGDLEGSGKHSS

γ2a RKVDSPFGPGSPSKGFFSRGPQPRPSSPMSAPVRPKTSPGSPKTVFPFSYQESPPRSPRR
γ2c RKVDSPFGPGSPSKGFFSRGPQPRPSSPMSAPVRPKTSPGSPKTVFPFSYQESPPRSPRR

γ2a MSFSGIFRSSSKESSPNSNPATSPGGIRFFSRSRKTSGLSSSPSTPTQVTKQHTFPLESY
γ2c MSFSGIFRSSSKESSPNSNPATSPGGIRFFSRSRKTSGLSSSPSTPTQVTKQHTFPLESY
γ2_3B MKRFGSLRSNKKHKDQNRSTERRQSEPHGLFASGLSSSPSTPTQVTKQHTFPLESY

γ2a KHEPERLENRIYASSSPPDTGQRFCPSSFQSPTRPPLASPTHYAPSKAAALAAALGPAEA
γ2c KHEPERLENRIYASSSPPDTGQRFCPSSFQSPTRPPLASPTHYAPSKAAALAAALGPAEA
γ2_3B KHEPERLENRIYASSSPPDTGQRFCPSSFQSPTRPPLASPTHYAPSKAAALAAALGPAEA

γ2a GMLEKLEFEDEAVEDSESGVYMRFMRSHKC
γ2c GMLEKLEFEDEAVEDSESGVYMRFMRSHKC
γ2_3B GMLEKLEFEDEAVEDSESGVYMRFMRSHKC
γ2b MLEKLEFEDEAVEDSESGVYMRFMRSHKC

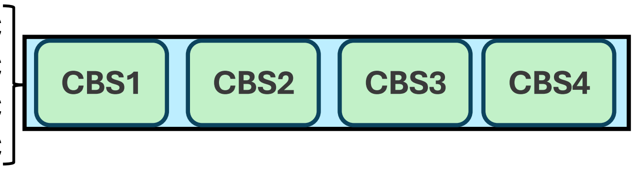

B

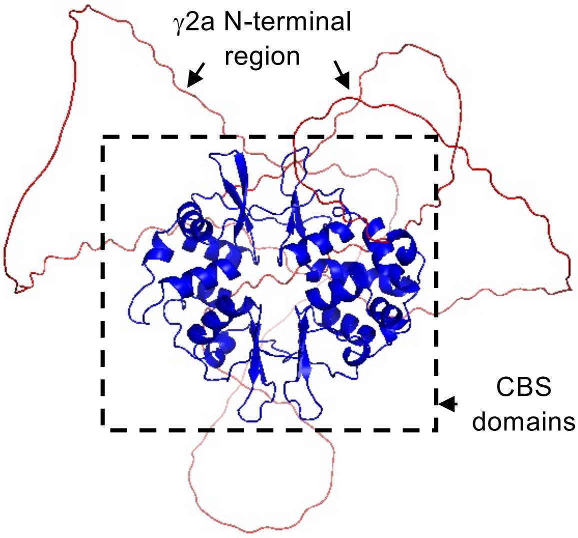

C

T97 S122 S131

Homo sapiens PMSAPVRPK**T**SPGSPKTVFPFSYQESPPRSPRRM**S**FSGIFRSSS**S**KESSPN
Pan troglodytes PMSAPVRPK**T**SPGSPKTVFPFSYQESPPRSPRRM**S**FSGIFRSSS**S**KESSPN
Sus scrofa PVSAPVRPK**T**SPGSPKTVFPFSYQESPPRSPRRM**S**FSGIFRSSS**S**KESSPS
Bos taurus PVSAPVRPK**T**SPGSPKTVFPFSYQESPPRSPRRM**S**FSGIFRSSS**S**KDSSPS
Mus musculus PVSAPVRPK**T**SPGSPKTVFPFSYQESPPRSPRRM**S**FSGIFRSSS**S**KESSPN
Rattus norvegicus PVSAPVRPK**M**SPGSPKTVFPFSYQESPPRSPRRM**S**FSGIFRSSS**S**KESSPS

Supplementary Figure S1. N terminal region of four γ2 transcript variants.

(A) Protein sequence alignment of the N-terminal regions of the four γ2 variants. The sequence of the C-terminal region, harbouring the four cystathionine-β-synthase (CBS) domains is identical in all four variants. (B) Predicted structure of γ2a by AlphaFold showing a disordered N-terminal region (in red) and the highly symmetrical C-terminal region harbouring the four CBS domains (in blue). (C) Sequence alignment of AMPKγ2a spanning T97, S122 and S131 (shown in bold red font) from the indicated species. Residues which are not conserved with the human sequence are denoted by blue text.

Supplementary Figure S2

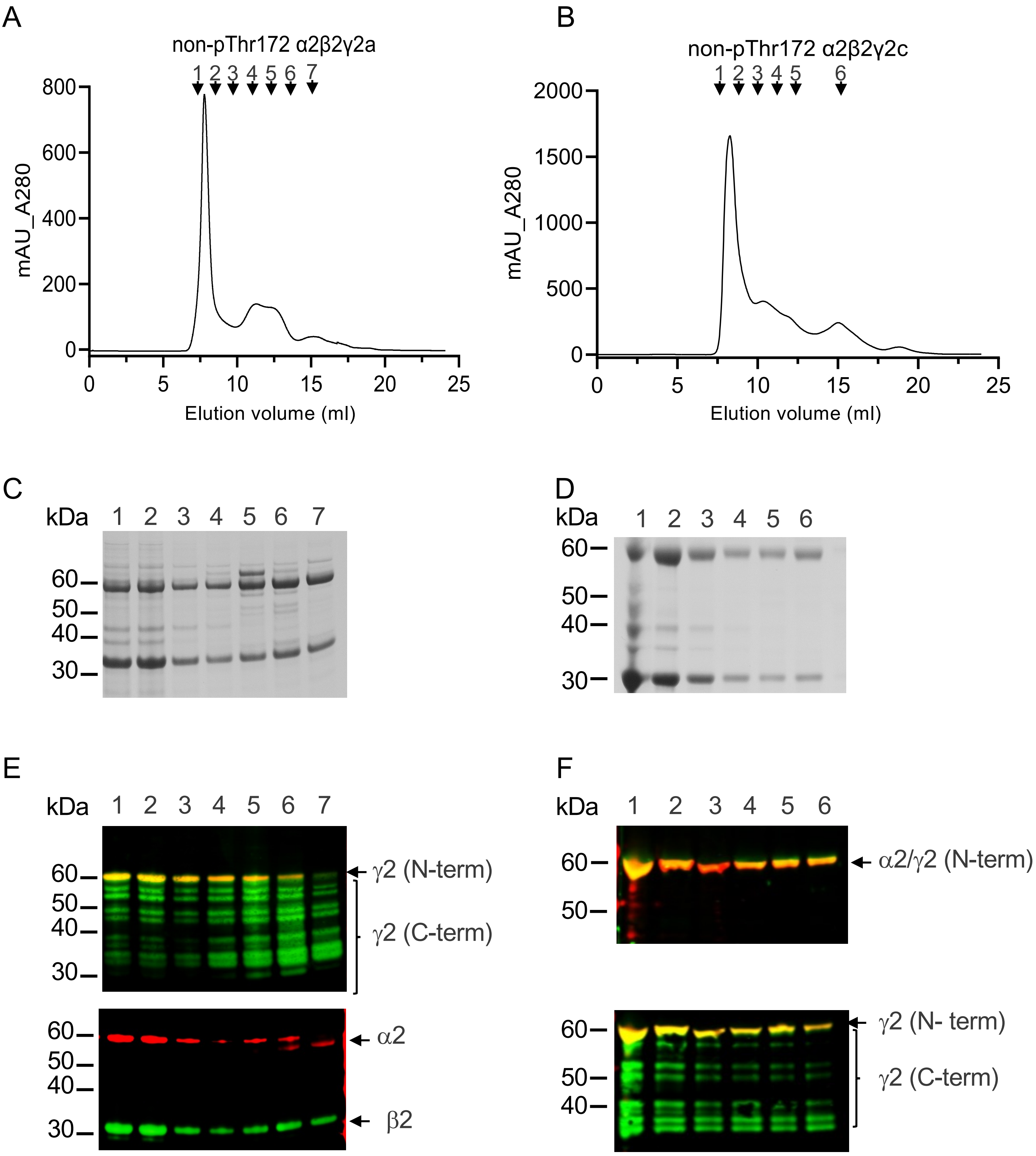

Supplementary Figure S2. Purification of recombinant AMPKγ2a/γ2c-containing complexes from bacteria.

AMPKα2β2γ2a and AMPKα2β2γ2c were expressed in bacteria and cell lysates purified by chromatography on a His-Trap column (the γ2 proteins harbour an N-terminal hexahistidine tag) followed by gel-filtration (Superdex200 10/300 column). (A, B) Elution profiles (absorption at 280 nm) following gel-filtration. (C, D) Coomassie-stained SDS-PAGE gel for the indicated fractions across the elution peaks. (E) Western blot analysis of the fractions following gel-filtration of AMPK α2β2γ2a. The top panel show a blot probed with an anti-His antibody detecting the N-terminus of γ2 (shown in the red channel) and an anti-γ2 C-terminal antibody (shown in the green channel). The bottom panel shows a separate blot probed with an anti-α2 antibody (red channel) and an anti-β2 antibody (green channel). (F) Western blot analysis of the fractions following gel-filtration of AMPK α2β2γ2c. The top panel show a blot probed with an anti-His antibody detecting the N-terminus of γ2 (shown in the red channel) and an anti-α2 antibody (shown in the green channel). The bottom panel shows the same blot re-probed with an anti-γ2 C-terminal antibody (shown in the green channel). The migration of molecular mass standards is shown on the left of the gels/blots and arrows identifying the bands corresponding to α2, β2, γ2a/c are indicated on the right of the blots.

Supplementary Figure S3

A

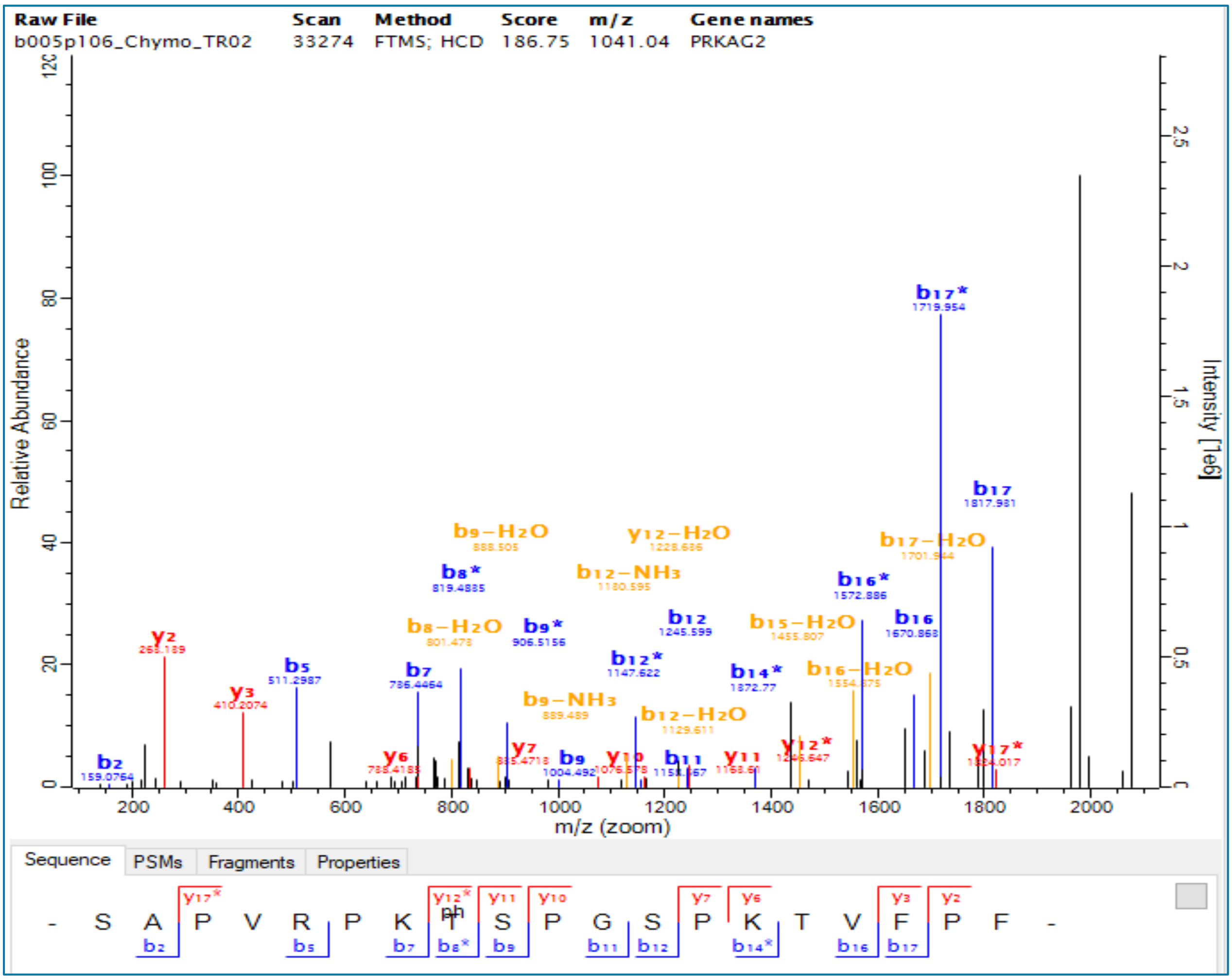

B

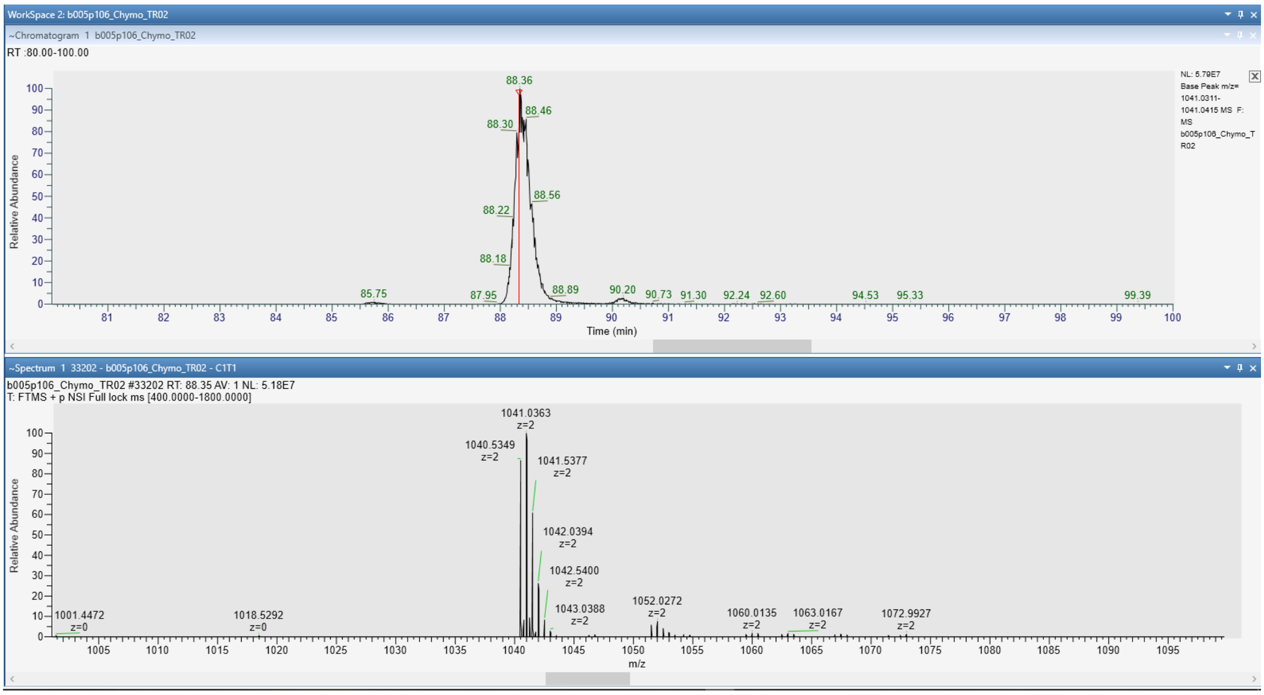

Supplementary Figure S3

C

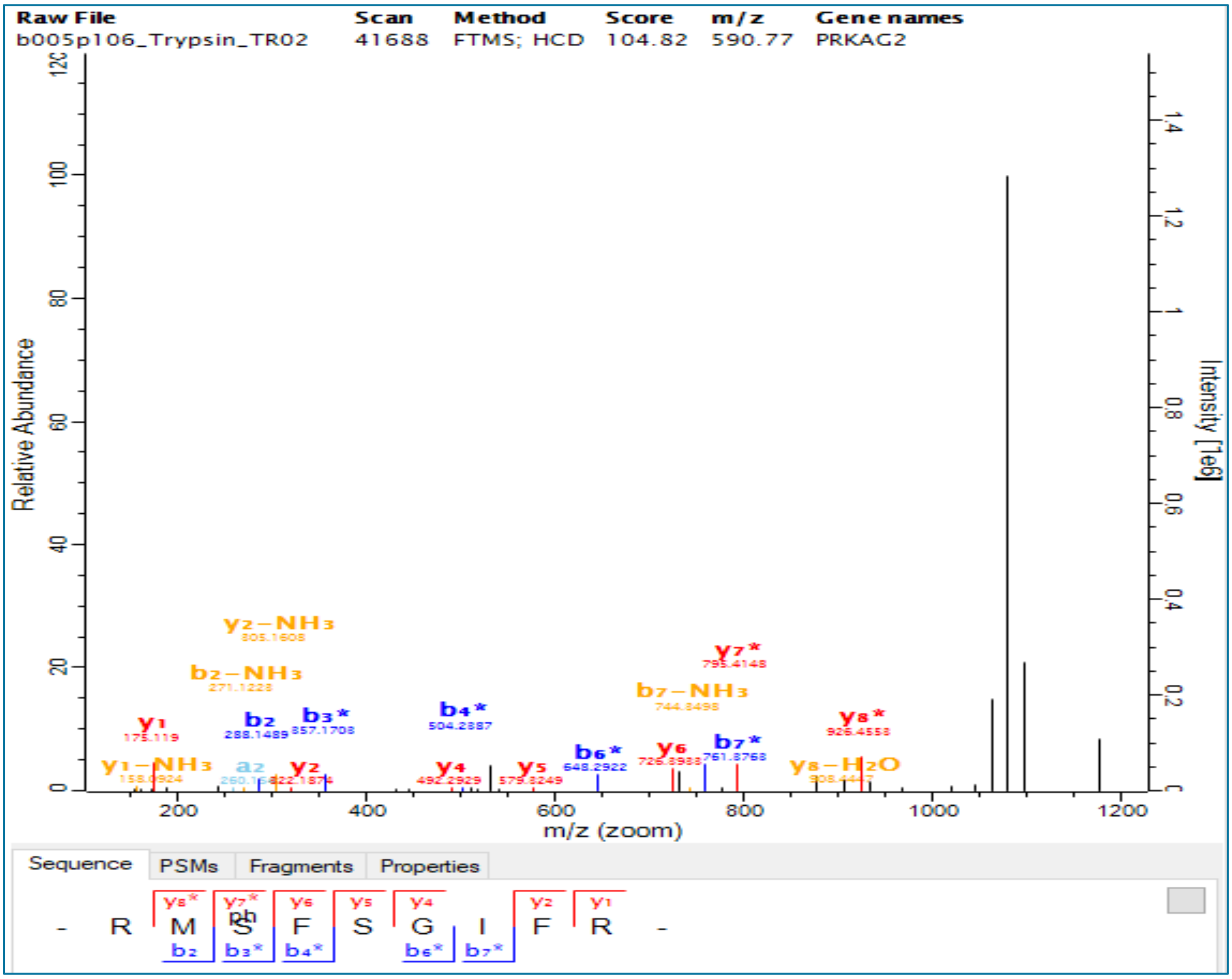

D

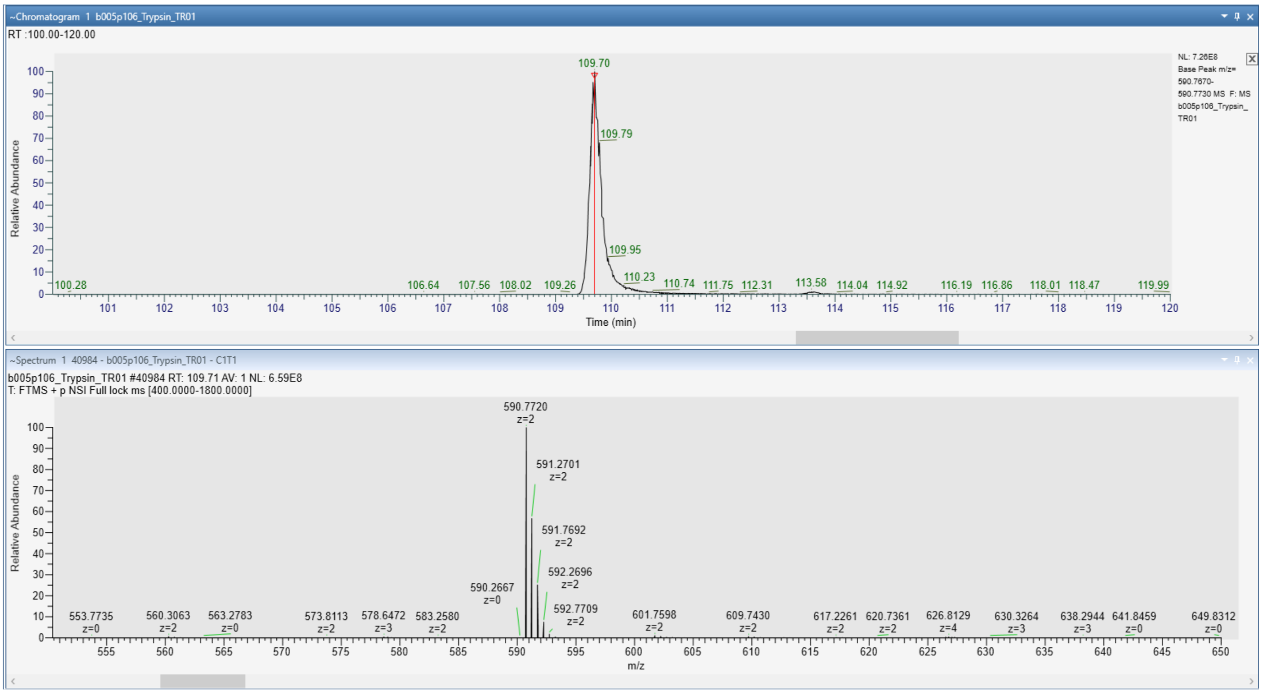

Supplementary Figure S3

E

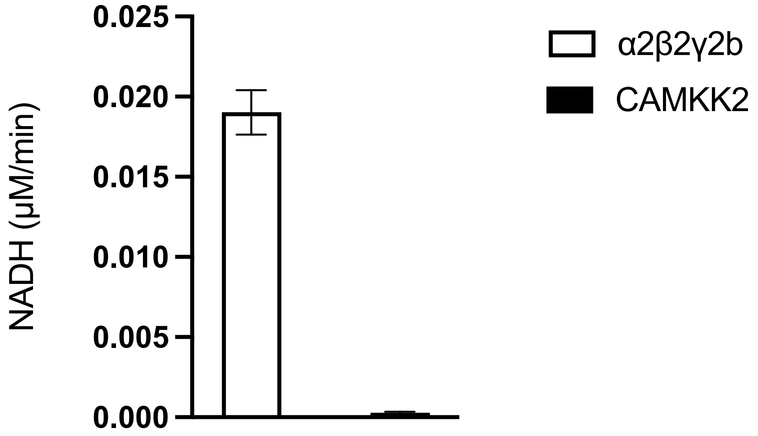

Supplementary Figure S3. Identification of pT53 and pS78 in recombinant AMPKα2β2γ2c-14-3-3ε complex.

Purified AMPKα2β2γ2c-14-3-3ε (2 μg) was digested with either chymotrypsin (A, B) or trypsin (C, D) and the resulting phosphopeptides analysed by mass spectrometry. The annotated fragmentation spectra analysed using MaxQuant (A, C) and the raw feature inspection obtained using Thermo Freestyle (B, D) are shown. (E) γ2a-T97-S122 (100 mM), the same peptide that was used for the isothermal calorimetry study, was incubated with either bacterially expressed recombinant AMPKα2β2γ2b (pThr172 complex) or CAMKK2. Peptide phosphorylation was measured using a coupled NADH assay. The rate of NADH utilisation (μM/min) was determined by measuring absorbance at 340 nm. Results shown are the mean ±SEM from 3 independent experiments.
